# ImputeHiFI: An Imputation Method for Multiplexed DNA FISH Data by Utilizing Single‐Cell Hi‐C and RNA FISH Data

**DOI:** 10.1002/advs.202406364

**Published:** 2024-09-12

**Authors:** Shichen Fan, Dachang Dang, Lin Gao, Shihua Zhang

**Affiliations:** ^1^ School of Computer Science and Technology Xidian University Xi'an 710071 China; ^2^ School of Automation Northwestern Polytechnical University Xi'an 710072 China; ^3^ NCMIS, CEMS, RCSDS Academy of Mathematics and Systems Science Chinese Academy of Sciences Beijing 100190 China; ^4^ School of Mathematical Sciences University of Chinese Academy of Sciences Beijing 100049 China; ^5^ Key Laboratory of Systems Biology Hangzhou Institute for Advanced Study University of Chinese Academy of Sciences Chinese Academy of Sciences Hangzhou 310024 China

**Keywords:** 3D genomes, single‐cell Hi‐C data, DNA FISH data, multimodal imaging, imputation

## Abstract

Although multiplexed DNA fluorescence in situ hybridization (FISH) enables tracking the spatial localization of thousands of genomic loci using probes within individual cells, the high rates of undetected probes impede the depiction of 3D chromosome structures. Current data imputation methods neither utilize single‐cell Hi‐C data, which elucidate 3D genome architectures using sequencing nor leverage multimodal RNA FISH data that reflect cell‐type information, limiting the effectiveness of these methods in complex tissues such as the mouse brain. To this end, a novel multiplexed DNA FISH imputation method named ImputeHiFI is proposed, which fully utilizes the complementary structural information from single‐cell Hi‐C data and the cell type signature from RNA FISH data to obtain a high‐fidelity and complete spatial location of chromatin loci. ImputeHiFI enhances cell clustering, compartment identification, and cell subtype detection at the single‐cell level in the mouse brain. ImputeHiFI improves the recognition of cell‐type‐specific loops in three high‐resolution datasets. In short, ImputeHiFI is a powerful tool capable of imputing multiplexed DNA FISH data from various resolutions and imaging protocols, facilitating studies of 3D genome structures and functions.

## Graphical abstract

ImputeHiFI utilizes the complementary structural information from single‐cell Hi‐C data and the cell‐type signatures from the RNA fluorescence in situ hybridization (FISH) data to obtain high‐fidelity and complete spatial locations of chromatin loci. ImputeHiFI consists of three steps, i.e., matching cells, estimating the values of loci pairs, and inferring the 3D coordinates of loci. ImputeHiFI can impute multiplexed DNA FISH data from various resolutions and imaging protocols.

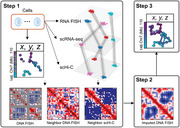

## 1 Introduction

Genomic DNA folds into complex 3D structures within cell nuclei. Understanding 3D genome structures is crucial for studying gene regulation, cell fate determination, and species evolution.^[^ ^1^, ^2^, ^3^, ^4^, ^5^ ^]^ Recent advances in single‐cell sequencing and imaging techniques have enhanced the ability to examine chromosomal structures at the single‐cell level. For instance, single‐cell Hi‐C (scHi‐C) has been applied to probe intercellular variability and dynamics of chromatin structure among cell populations or across some critical biological processes.^[^ ^6^, ^7^, ^8^, ^9^, ^10^, ^11^, ^12^, ^13^ ^]^

In parallel, multiplexed DNA fluorescence in situ hybridization (FISH) based imaging technologies initially design specific DNA probes for each targeted genomic locus and label these loci with fluorescent probes of different colors. Subsequent rounds of hybridization and imaging are conducted, and the results from these multiple imaging rounds are combined to obtain spatial locations for hundreds to thousands of genomic loci.^[^ ^14^, ^15^, ^16^ ^]^ The whole chromatin imaging provides direct visualization of the 3D genomic organization of individual cells in the context of the structure and function of the cell nucleus.^[^ ^17^, ^18^, ^19^, ^20^, ^21^, ^22^, ^23^, ^24^, ^25^ ^]^ Jia et al. developed a spatial genome aligner that extracts 3D spatial coordinates from the multiplexed DNA FISH signals.^[^ ^26^ ^]^ They could estimate the spatial distance between two genomic loci by a Gaussian chain model. Subsequently, they compared this estimated distance with the observed one, thereby assessing the physical probability of the observed loci signals and constructing the chromatin structure. Devos et al. developed pyHiM to analyze spatial genomics data from multiplex DNA FISH imaging.^[^ ^27^ ^]^ pyHiM can reconstruct chromatin structures at the single‐cell level from original 3D imaging data and integrates functionalities for image projection, segmentation, matching, and post‐processing analysis. However, multiplexed DNA FISH experiments involve multiple rounds of hybridization and imaging. Technical limitations lead to the non‐detection of many probes' imaging signals. We refer to them as missing probes. Typically, the missing rate for probes in individual cells can be as high as 25%,^[^ ^28^ ^]^ and it may reach 62%.^[^ ^22^, ^23^ ^]^ This substantial missing severely compromises the completeness of the genomic information and the reliability of downstream analyses. Consequently, developing an effective imputation method to address these gaps in multiplexed DNA FISH data is critically important.

Two imputation methods have been proposed to address missing loci in multiplexed DNA FISH data. Linear interpolation estimates the 3D coordinates of missing positions by calculating a weighted average between two adjacent locations.^[^ ^28^ ^]^ SnapFISH‐IMPUTE^[^ ^29^ ^]^ incorporates information from adjacent positions and chromosomal conformation observed in other single cells with similar structures. SnapFISH‐IMPUTE uses a similarity metric to identify chromosomes with analogous conformations, constructs a target paired distance matrix based on the chosen structure, and minimizes discrepancies between this matrix and the spatial distances derived from the 3D coordinates. SnapFISH‐IMPUTE significantly enhances imputation accuracy in cases of extensive data missingness. However, in complex tissues that contain multiple cell types, relying solely on single‐cell data from a single modality is often insufficient to depict the cellular state. Current imputation methods do not fully exploit the information from multimodal (same cell) and multi‐omics (same tissue) data, which limits their effectiveness in addressing complex tissues such as the mouse brain.

The advancement of experimental techniques has yielded several multimodal and multi‐omics imaging and sequencing data. For imaging data, Su et al. designed a whole‐genome chromatin tracking method based on DNA multiplexed error‐robust FISH (MERFISH),^[^ ^30^ ^]^ which images over 1000 genomic loci and nascent transcripts of over 1000 genes as well as landmark cell nuclear structures in human lung fibroblast (IMR‐90) cells. Huang et al.^[^ ^28^ ^]^ employed multimodal DNA MERFISH and RNA MERFISH techniques to measure the 3D chromatin structure of the Sox2 gene locus and the specific transcriptional activity at this locus in mouse embryonic stem cells (mESCs). Takei et al.^[^ ^22^ ^]^ presented the imaging results of 3660 chromosomal loci and 70 RNAs in individual mESCs using DNA seqFISH+ and RNA seqFISH. Takei et al.^[^ ^23^ ^]^ imaged 3660 DNA loci and 76 RNAs in the adult mouse cerebral cortex cells. Liu et al.^[^ ^25^ ^]^ utilized multimodal DNA MERFISH and RNA MERFISH techniques to measure 1981 DNA loci and 242 RNAs in single cells from the mouse primary motor cortex. For sequencing data, Tan et al. used MALBAC‐DT^[^ ^31^ ^]^ and Dip‐C^[^ ^12^ ^]^ to generate 3D genome structures and transcriptomes of the developing mouse cortex and hippocampus after birth, which increases understanding of the dynamics of the postnatal single‐cell transcriptome and 3D genome.^[^ ^32^ ^]^ Tan et al. utilized the Dip‐C technique to elucidate the genome structure of the mouse retina and main olfactory epithelium.^[^ ^13^ ^]^ Additionally, Tan et al. sequenced the 3D genome structures and transcriptomes of cerebellar granule cells in humans (ranging from 0.1 to 86 years old) and mice (from birth to 21 months) to study the changes in chromosomal structure and gene expression throughout their lifespans.^[^ ^33^ ^]^ Liu et al. used HiRES (Hi‐C and RNA‐seq Employed Simultaneously) technology to sequence mouse embryonic single cells at various developmental stages, ranging from embryonic day 7.0 to 11.5.^[^ ^34^ ^]^ Tian et al. analyzed DNA methylation and chromatin conformation in 399 000 neurons and 118 000 non‐neuronal cells across 46 regions of the brains of three adult males, constructing a multimodal epigenetic atlas of the human brain.^[^ ^35^ ^]^ Wu et al. proposed a technology LiMCA (Linking mRNA to Chromatin Architecture) that simultaneously sequences the 3D genome structures and complete transcriptomes of single cells with high sensitivity. They utilized this technology to sequence mouse main olfactory epithelial cells at various time points, aiming to investigate the developmental processes of olfactory sensory neurons.^[^ ^36^ ^]^ RNA FISH data expose the wide variety of cell types within tissues. Su et al. and Takei et al. demonstrated high consistency between Hi‐C and multiplexed DNA FISH data. For instance, the proximity scores derived from imaging data exhibited a Spearman correlation coefficient exceeding 0.83 with Hi‐C data.^[^ ^21^, ^22^, ^23^ ^]^ Therefore, the abundant multimodal and multi‐omics data generated by current experimental technologies provide a robust resource for imputing missing data in multiplexed DNA FISH techniques.

In this work, we present a method ImputeHiFI for imputing the multiplexed DNA FISH data by utilizing scHi‐C and RNA FISH data. Compared to existing ones, ImputeHiFI notably enhances the utility of multiplexed DNA FISH data in downstream analyses such as clustering, compartment identification, cell subtype detection, and loop detection. In short, ImputeHiFI is the first approach to utilize multimodal and multi‐omics information for the imputation of multiplexed DNA FISH data, thereby significantly advancing the study of genome architecture and expression at the single‐cell level.

## 2 Results

### 2.1 Elucidating the Rationale for Utilizing Similar Chromosomal Structures, RNA FISH, and scHi‐C Data to Impute Multiplexed DNA FISH Data

**Figure 1:**
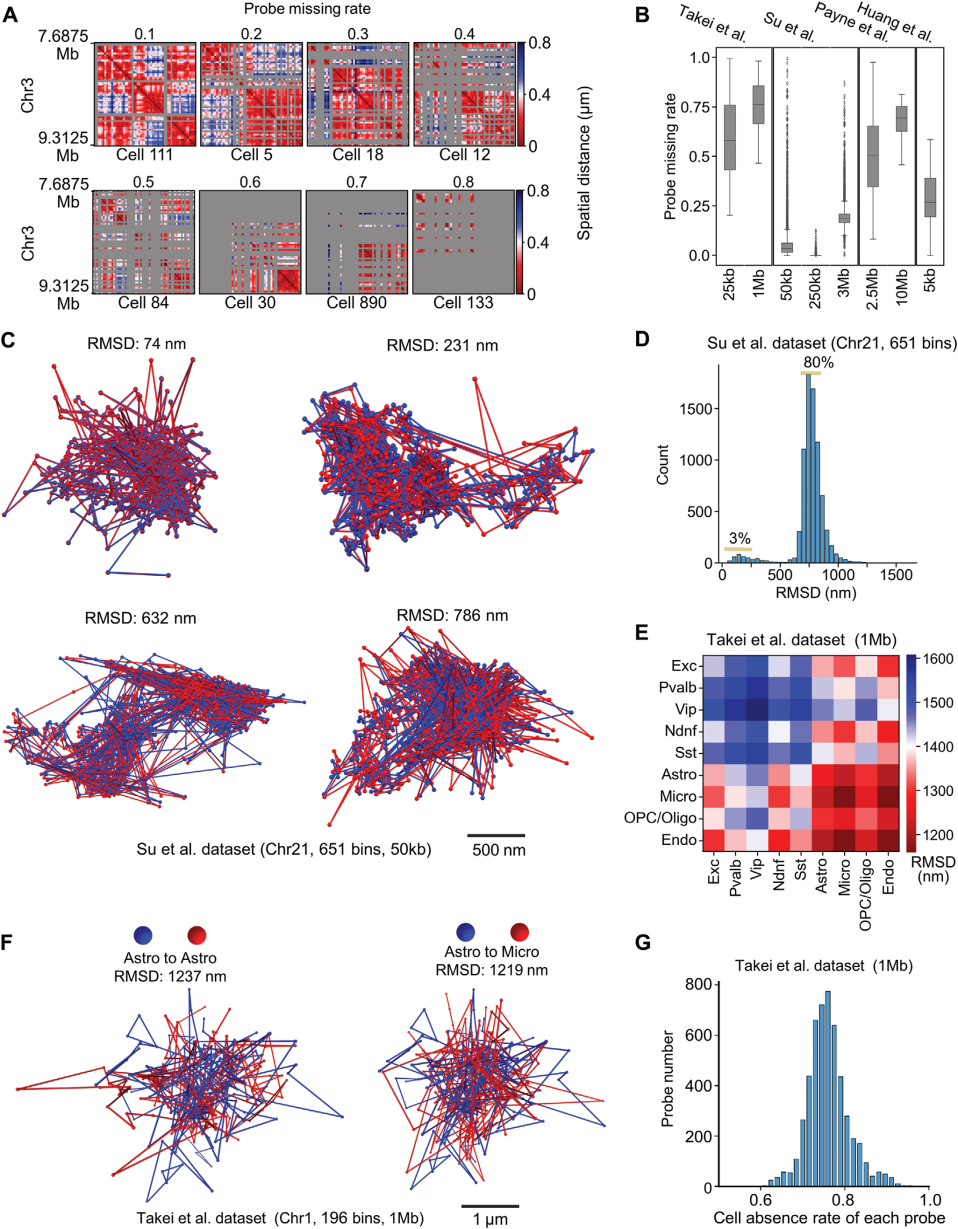
The probe missing rates and chromosomal structural similarities in multiplexed DNA FISH datasets. A) Heatmap of single‐cell spatial distances with varying probe missing rates for the mouse brain dataset at the resolution 25Kb (Takei et al., 2021). The probe missing rate is quantified as the ratio of the bin number without probe signals to the total bin number in the chromosomal region. The cell names are displayed below the heatmap. B) The probe missing rates of Takei et al., Su et al., Payne et al., and Huang et al. dataset, respectively. The probe missing rate quantifies the proportion of undetected probes relative to the total number of probes within a cell. C) 3D visualization of the imaging data at the resolution of 50 kb on chromosome 21. Red and blue represent chromosome 21 from different cells or copies, respectively. D) Distribution of the RMSD values for each chromosome relative to its nearest neighboring chromosome (Su et al. dataset, chr21, 50 kb, 651 bins). The percentage of RMSD values from 0 to 250 is 3% (278 of 7967), and from 600 to 800 is 80% (6352 of 7967). The neighbor bin distance is 500 nm. E) RMSD values between the different cell types of 1Mb resolution (Takei et al. mouse brain dataset). F) 3D visualization of two cells’ probe coordinates from Takei et al. brain dataset: Left: Astro (cell name ‘1354′) to Astro (cell name ‘1170′); Right: Astro (cell name ‘1354′) to Micro (cell name ‘1324′). G) Distribution of probe numbers with different cell absence rates of each probe. For each probe, the cell absence rate is the number of cells in which it was undetected, divided by the total number of cells. The mean cell absence rate of each probe is 0.76. The minimum is 0.6.

To demonstrate the rates of missing probes, we analyzed the multiplexed DNA FISH data from Takei et al. on mouse brain cells^[^ ^23^ ^]^ and mESCs,^[^ ^22^ ^]^ Su et al. on IMR90 cells,^[^ ^21^ ^]^ Payne et al. on early mouse embryo cells^[^ ^20^ ^]^ and Huang et al. on mESCs.^[^ ^28^ ^]^ We found that the average missing probe rate ranged from 5% to 75% (**Figure** **1**B). Furthermore, we presented spatial distance matrices at different missing probe rates. When the probe missing rate reached 80%, the interaction patterns of chromosomes were almost completely lost (Figure 1A).

To assess whether chromosomes of the same cell type exhibit similar structures utilized to impute missing data, we selected the chr21 dataset provided by Su et al. specifically for IMR90 cells. This dataset contains 651 bins and 7967 chromosomes at a resolution of 50 kb. Notably, with an average probe missing rate of only 5% (Figure 1B), this dataset provides a stable foundation for subsequent analyses. To precisely measure the differences in chromosome structures, we employed the Root Mean Square Deviation (RMSD) statistical method, which quantitatively describes the variability of 3D structures (details in Section 5.4.1). For each chromosome, we search for the most similar one with structure and record the RMSD between them (Figure 1D). We noticed that 3% of the most identical chromosome pairs had RMSD values between 0 and 250 nm, and 80% had RMSD values between 600 and 800 nm. 3D visualization of chromosomes reveals that when the RMSD value is between 0 and 250 nm, the structures of the two chromosomes are identical. When the RMSD value ranges from 600 to 800 nm, the large‐scale frameworks of the two chromosomes are similar (Figure 1C). Overall, we can leverage the information from chromosomes with similar structures to impute missing data.

Next, we utilized the mouse brain multiplexed DNA FISH dataset provided by Takel et al. at a 1Mb resolution to explore whether RMSD varies among different cell types. We found that the RMSD within the same cell type was not significantly smaller than that between different cell types (Figure 1E). For instance, the RMSD between Astro and Astro was even higher than that between Astro and Micro (Figure 1E,F). We observed that the cell absence rate for each probe in multiplexed DNA FISH data exceeded 60% (Figure 1G). Such extensive data loss may hinder the ability of RMSD to reflect the true cell types. Current multimodal imaging techniques enable the simultaneous imaging of both DNA FISH and RNA FISH data within the same cell. RNA FISH data can provide complementary information to identify cell types (Figure S1B, Supporting Information). By clustering cells based on RNA FISH data and constructing a K‐nearest neighbors (KNN) graph based on RMSD within these clusters, we can merge the DNA FISH data of adjacent cells. This merging mitigates the effects of data loss, enabling the multiplexed DNA FISH data to display different cell types (Figure S1C, Supporting Information). In summary, RNA FISH data act as auxiliary information to indicate cell type, ensuring that cells within a cluster consist of similar types. For a cell with missing DNA FISH data, we identified other cells with similar chromosomal structures from the same cell types and utilized their chromosomal structure information to impute the missing DNA FISH data.

**Figure 2:**
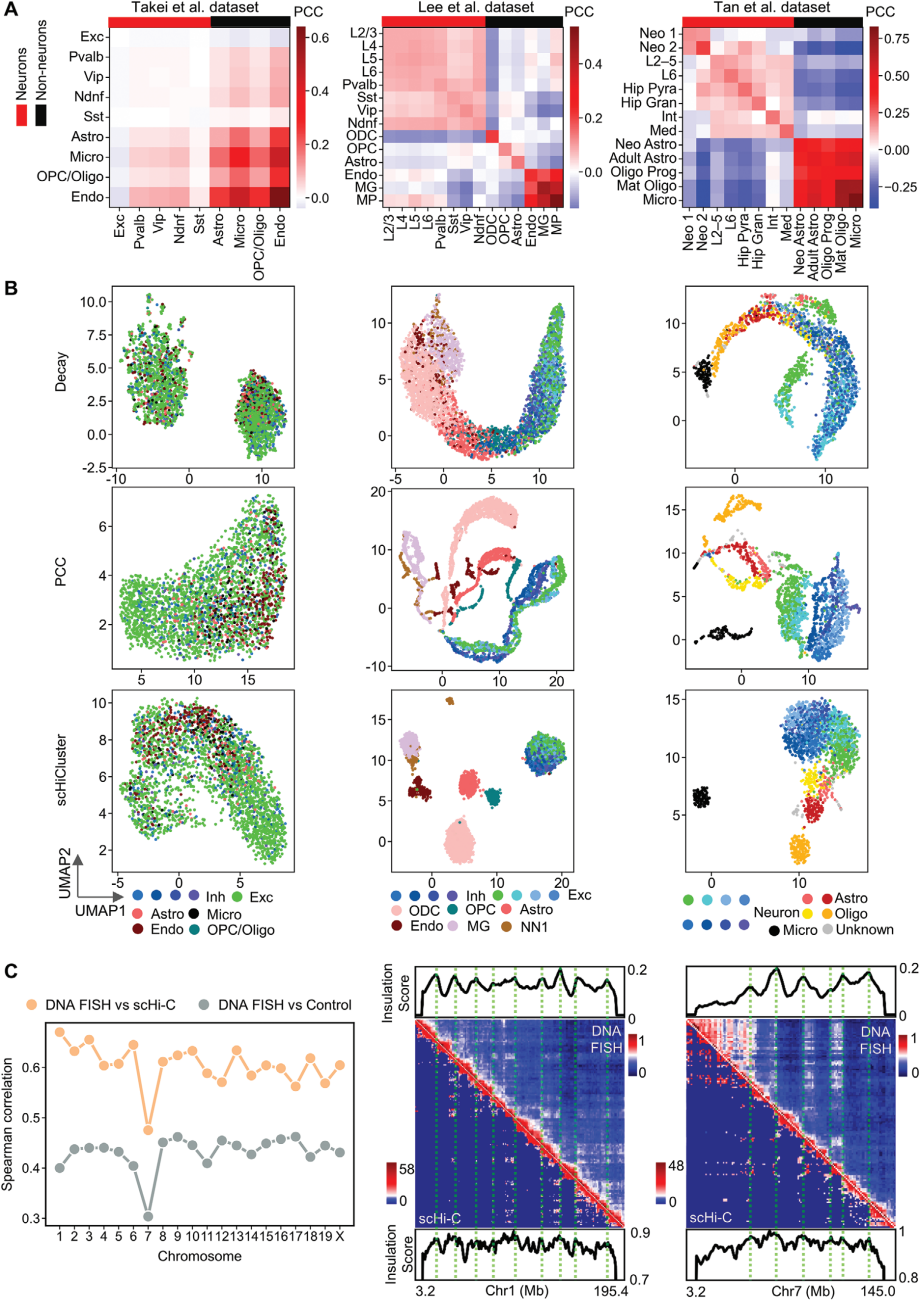
The clustering results of multiplexed DNA FISH data (Takei et al. mouse brain data at the resolution 1Mb) and single‐cell Hi‐C data (Lee et al. human brain data and Tan et al. mouse brain data). A) Pearson correlation coefficient within and between cell types for single cells. Each cell uses locus pairs with high variability as features. The variability of locus pairs is defined by the coefficient of variation, i.e., the standard deviation divided by the mean. B) UMAP visualization of three datasets with three different clustering methods. For the Takei et al. dataset, Exc means excitatory neurons. Inh means inhibitory neurons, which includes ‘Pvalb’, “Sst”, “Vip”, and “Ndnf”. OPC/Oligo, oligodendrocyte progenitor cells, and oligodendrocytes. For the Lee et al. dataset, “Exc” means excitatory neurons, which include “L2/3′, ‘L4”, “L5”, and “L6”. “Inh” means inhibitory neurons, which include “Pvalb”, “Sst”, “Vip”, and “Ndnf”. “Astro”, astrocyte. “ODC”, oligodendrocyte. “OPC”, oligodendrocyte progenitor cell. “MG”, microglia. “NN1”, non‐neuronal cell type 1. “Endo”, endothelial cell. For the Tan et al. dataset, Neuron type includes neonatal neuron 1, neonatal neuron 2, cortical l2–5 pyramidal cell, cortical l6 pyramidal cell, hippocampal pyramidal cell, hippocampal granule cell, interneuron, medium spiny neuron, astrocyte type includes neonatal astrocyte, adult astrocyte. Oligodendrocyte type includes oligodendrocyte progenitor and mature oligodendrocyte. C) Agreement between the merged 1Mb proximity score maps from multiplexed DNA FISH data and the merged 1Mb chromatin interaction maps from scHi‐C data for excitatory neurons (left). Control data are multiplexed DNA FISH data of other cell types. scHi‐C data (322 cells) are displayed with 1Mb bin size to compare with 1Mb resolution multiplexed DNA FISH data (1895 cells) for excitatory neurons (chr1 and chr7, middle and right, respectively). The vertical green lines on the heatmaps indicate the positions of local maxima of the insulation score, where interactions between chromosomal segments on either side are typically reduced.

To assess the capability of scHi‐C data in revealing cell types within complex tissues, we analyzed the clustering effects of two distinct scHi‐C datasets and compared the results with that of a multiplexed DNA FISH dataset. Lee et al.^[^ ^37^ ^]^ obtained single‐cell Hi‐C data for 14 cell types from the human frontal cortex, with cell type labels provided by clustering cytosine DNA methylation data. Tan et al.^[^ ^32^ ^]^ obtained scHi‐C data for 13 cell types from the mouse cortex and hippocampus, with cell type labels derived by cross‐referencing with single‐cell RNA sequencing (scRNA‐seq) data. Takei et al.^[^ ^23^ ^]^ obtained multiplexed DNA FISH data for 9 cell types from the mouse cerebral cortex, with cell type labels assigned by clustering RNA FISH data. For the multiplexed DNA FISH data, we first calculated the spatial distances between each two chromosomal segments to obtain the spatial distance matrix (details in Section 5.2). Following Takei et al., we employed a Gaussian kernel function to transform the spatial distance matrix into the proximity score matrix (details in Section 5.3).^[^ ^23^ ^]^ A higher proximity score indicates a closer spatial distance between chromosomal segments. We used three clustering methods (Decay,^[^ ^8^ ^]^ PCC,^[^ ^38^ ^]^ scHiCluster^[^ ^39^ ^]^) on the three brain datasets as described above. The brain's cortical tissue contains neurons and non‐neurons. Neurons comprise excitatory and inhibitory types, while non‐neurons include many subtypes such as astrocytes and endothelial cells. We noticed a clear difference between neuronal and non‐neuronal cells in two scHi‐C datasets (from Lee et al. and Tan et al.) by calculating the Pearson correlation coefficient between cells, respectively. However, this clear boundary between the two major types is not observed on the multiplexed DNA FISH dataset (**Figure** **2**A). We then visualized the clustering results of the three methods with UMAP. Despite the differences in the clustering effects of the three methods, the same types of cells form clear clusters on the scHi‐C data, while different types of cells are mixed on the multiplexed DNA FISH dataset (Figure 2B). The success of Decay in clustering the scHi‐C data indicates that the ratios of interactions across different genomic distances can distinguish different cell types. PCC successfully classified scHi‐C data using highly variable locus pairs. The clustering results of scHiCluster indicate that after using linear convolution and random walk for imputation, different cell types can be separated in the scHi‐C data. In short, scHi‐C data capture a wealth of information relevant to various cell types.

Then, we demonstrated a high level of agreement between the multiplexed DNA FISH data and scHi‐C data, consistent with Takei et al.’s findings.^[^ ^23^ ^]^ To compare scHi‐C and multiplexed DNA FISH data, we first calculated the median chromatin interaction matrices and proximity score matrices for multiple cells of the same type (Figures S2 and S3, Supporting Information). Subsequently, we noted that the Spearman correlation coefficient between multiplexed DNA FISH data and scHi‐C data within identical cell types was significantly higher than that between multiplexed DNA FISH data from different cell types (Figure 2C; Figure S4, Supporting Information). We also observed a similar trend in the boundary insulation score between multiplexed DNA FISH and scHi‐C data (Figure 2C). On the other hand, multiplexed DNA FISH data do not accurately reflect the differences between cell types (Figure 2A,B), potentially due to limitations in current imaging techniques, such as the absence of probes, insufficient probe resolution, and inaccurate spatial location of loci due to noise.^[^ ^26^ ^]^ In summary, scHi‐C data provides rich information about cell types and shows high concordance with multiplexed DNA FISH data, making it an effective resource for imputing missing data.

### 2.2 Overview of ImputeHiFI

**Figure 3:**
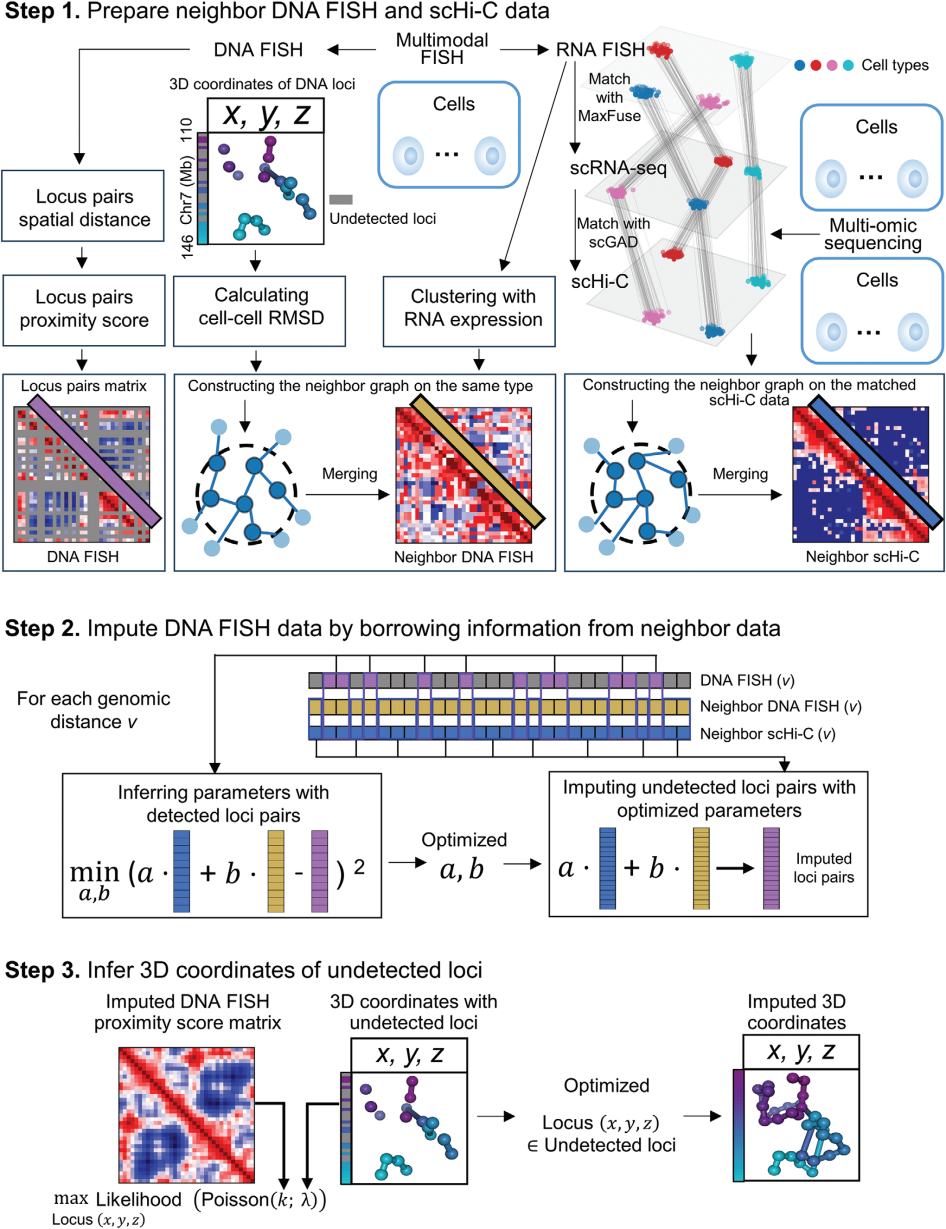
Overview of ImputeHiFI mode 1. To simplify our description, we referred to multiplexed DNA FISH as DNA FISH. Step 1: Prepare neighbor DNA FISH and scHi‐C data. ImputeHiFI uses multimodal DNA FISH, RNA FISH data, and multi‐omics scRNA‐seq and scHi‐C data. First, ImputeHiFI clusters the RNA FISH data, calculates the RMSD for DNA FISH on the same type of cells to build a cell‐type‐specific neighbor graph, and merges the neighbor DNA FISH data. Next, ImputeHiFI uses MaxFuse to match RNA FISH with scRNA‐seq data and scGAD to match scRNA‐seq with scHi‐C data. ImputeHiFI then creates a neighbor graph for the matched scHi‐C data and merges the neighbor scHi‐C data. Step 2: Impute the proximity score matrix of DNA FISH data by borrowing information from neighbor data. For each genomic distance *v*, ImputeHiFI determines neighbor scHi‐C data weight *a* and neighbor DNA FISH data weight *b* through the detected locus pairs in DNA FISH data. Then, ImputeHiFI uses *a* and *b* to impute the undetected locus pairs in DNA FISH data. Step 3: Infer 3D coordinates of undetected loci. Utilizing the imputed DNA FISH proximity score matrix obtained from step two, along with 3D coordinates containing undetected loci, ImputeHiFI models the proximity score as independent Poisson random variables *k*, where the 3D coordinates of loci serving as the Poisson parameter λ. By maximizing the likelihood, ImputeHiFI imputes the 3D coordinates of undetected loci.

We developed ImputeHiFI to impute multiplexed DNA FISH data by utilizing scHi‐C and RNA FISH data. To accommodate different data scenarios, ImputeHiFI is designed with three modes (**Table** **1**). Mode 1 combines multimodal multiplexed DNA FISH and RNA FISH with multi‐omics scHi‐C and scRNA‐seq data (**Figure** **3**). Mode 2 solely uses multimodal multiplexed DNA FISH and RNA FISH data (Figure S5, Supporting Information). Mode 3 relies exclusively on multiplexed DNA FISH data (Figure S6, Supporting Information). In this section, we outline the procedure for ImputeHiFI mode 1. Detailed descriptions of the three modes can be found in the experimental section.

**Table 1:** Datasets used for three modes of ImputeHiFI.

	DNA FISH	RNA FISH	scRNA‐seq	scHi‐C
ImputeHiFI mode 1	✓	✓	✓	✓
ImputeHiFI mode 2	✓	✓		
ImputeHiFI mode 3	✓			

✓: Dataset required

**Figure 4:**
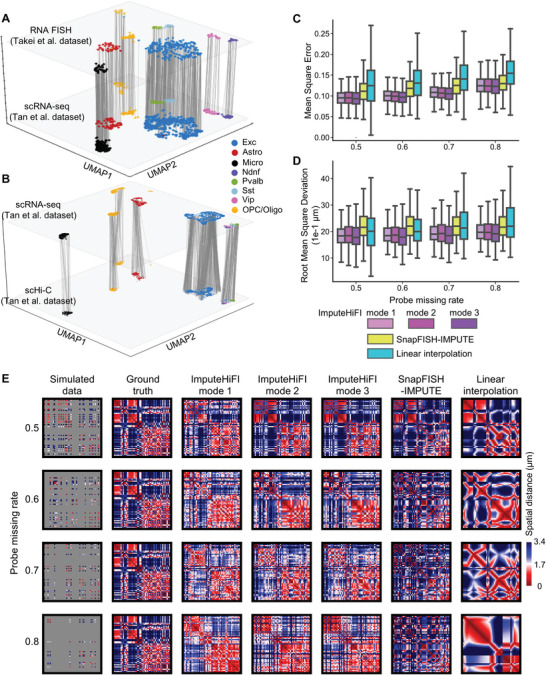
(A, B) Data preparation of ImputHiFI. (C, D, E) The imputation results of simulated multiplexed DNA FISH data. A) Match Takei et al. RNA FISH and Tan et al. scRNA‐seq data with MaxFuse. B) Match Tan et al. scRNA‐seq and Tan et al. scHi‐C data with scGAD. C) The performance of five imputation methods on the simulated dataset using MESE as the metric. D) The performance of five imputation methods on the simulated dataset using RMSD as the metric. E) Spatial distance heatmap of simulated data, ground truth, and the results of five imputation methods. Cell name: ‘1846′. Chr6: 50–100 Mb.

ImputeHiFI mode 1 consists of three steps. The first step involves the preparation of neighbor multiplexed DNA FISH and scHi‐C data. Initially, ImputeHiFI clusters RNA FISH data and calculates the RMSD for multiplexed DNA FISH on the same cell types. Then, ImputeHiFI constructs a neighbor graph for specific cell types and merges structurally similar DNA FISH data. Subsequently, ImputeHiFI uses MaxFuse^[^ ^40^ ^]^ to match RNA FISH with scRNA‐seq data (**Figure** **4**A; Figure S7A, Supporting Information) and employs scGAD^[^ ^41^ ^]^ to align scRNA‐seq with scHi‐C data (Figure 4B; Figure S7B, Supporting Information). Notably, although merged scHi‐C data and merged DNA FISH data show a high degree of consistency across the same cell types (Figure 2C), directly matching individual DNA FISH cells with scHi‐C cells is highly challenging due to significant missing loci in the DNA FISH data. Therefore, ImputeHiFI uses RNA FISH and scRNA‐seq data as a bridge to establish cell pairing between multiplexed DNA FISH and scHi‐C data. According to this, ImputeHiFI creates a neighbor graph for the matched scHi‐C data and merges neighboring scHi‐C data.

The second step involves imputing the proximity score matrix of multiplexed DNA FISH by leveraging information from neighboring multiplexed DNA FISH and scHi‐C data. In single‐cell Hi‐C data, the interactions between chromosomes notably diminish as the genomic distance increases. Similarly, multiplexed DNA FISH data reveal that greater genomic distances correlate with larger spatial separations. Therefore, individual optimization for different genomic distances can mitigate the interaction discrepancies caused by varying genomic distances. For each genomic distance *v*, ImputeHiFI employs the least square estimation to determine the weights *a* for neighboring scHi‐C data and *b* for neighboring multiplexed DNA FISH data, based on detected locus pairs in the multiplexed DNA FISH data. Then, ImputeHiFI uses *a* and *b* to impute the undetected locus pairs.

The third step involves inferring the 3D coordinates of undetected loci. Using the imputed multiplexed DNA FISH proximity score matrix obtained from the second step, along with the 3D coordinates containing undetected loci, ImputeHiFI models the proximity score as a Poisson random variable *k*, where the 3D coordinates of loci act as the Poisson parameter λ.^[^ ^42^ ^]^ By maximizing the likelihood, ImputeHiFI estimates the 3D coordinates of the undetected loci.

### 2.3 ImputeHiFI Performs Well on Simulated Multiplexed DNA FISH Data

To evaluate the imputation performance of different methods, we generated simulated datasets with probe missing rates of 50%, 60%, 70%, and 80% using the 1Mb dataset from Takei et al. on the mouse brain.^[^ ^23^ ^]^ We used two metrics for evaluation: Mean Squared Error (MSE) and RMSD. MSE is employed to quantify the differences between two chromosome structures (details in Section 5.4.2). A lower MSE indicates smaller differences between the chromosome structures. We used MSE to compare the imputed proximity score matrix and RMSD to compare the spatial 3D positions of loci after imputation. We assessed the imputation effectiveness of the three methods: ImputeHiFI, SnapFISH‐IMPUTE, and Linear interpolation. ImputeHiFI mode 1 utilized Tan et al.’s scHi‐C and scRNA‐seq data.^[^ ^32^ ^]^ ImputeHiFI mode 1 and mode 2 utilized Takei et al.’s RNA FISH data (Figure 4A,B).^[^ ^23^ ^]^ We observed that as the missing rate increased, the imputation errors for ImputeHiFI, SnapFISH‐IMPUTE, and Linear interpolation also increased; however, ImputeHiFI consistently showed lower MSE and RMSD values (Figure 4C,D). We visualized the heatmap of the imputed data, where ImputeHiFI restored the original chromosomal structures more effectively compared to SnapFISH‐IMPUTE and Linear interpolation (Figure 4E). Overall, ImputeHiFI outperforms existing imputation methods on simulated data.

### 2.4 ImputeHiFI Improves Clustering, Compartment Detection, and Cell Subtype Identification

**Figure 5:**
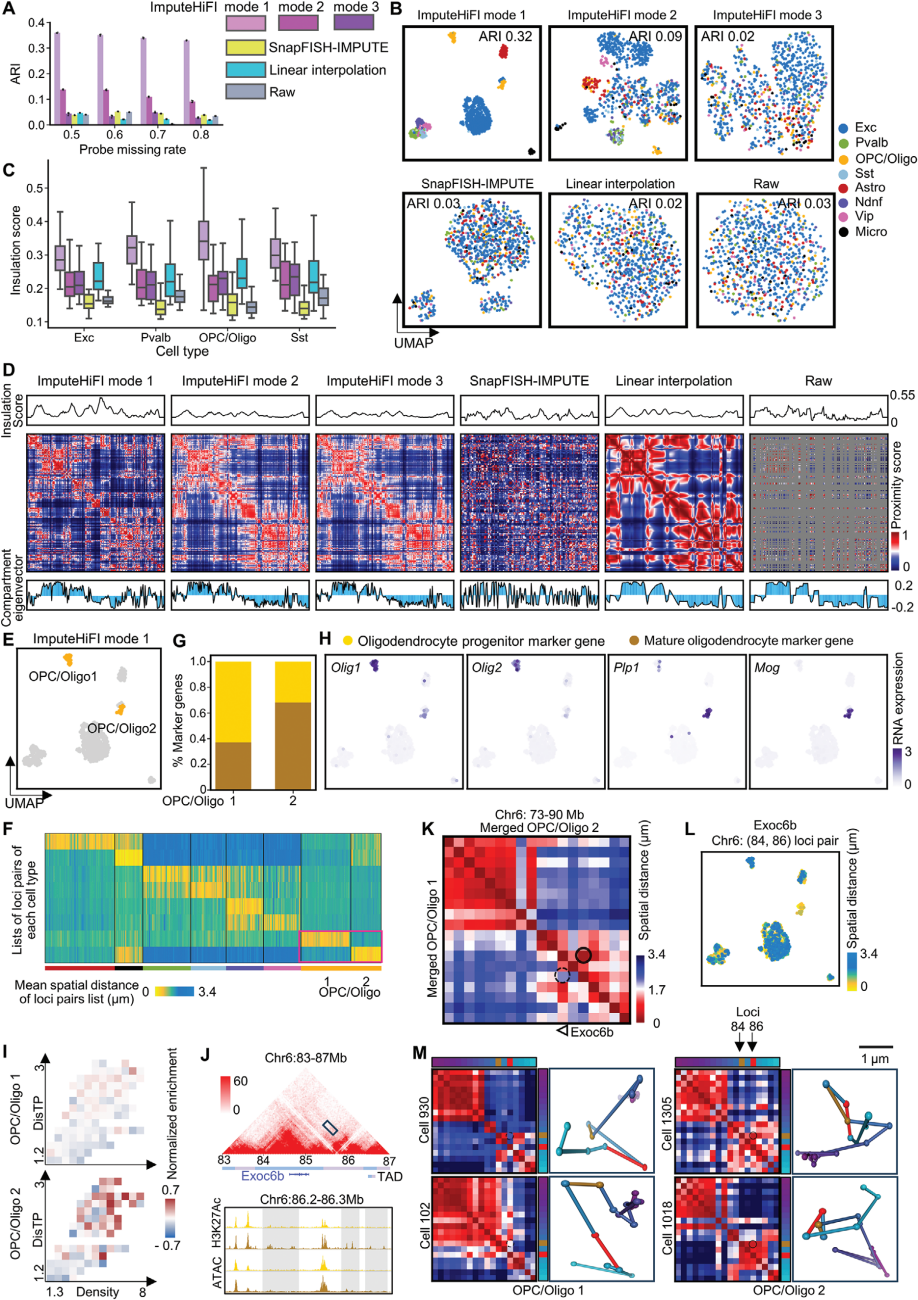
The imputation results of Takei et al. multiplexed DNA FISH data at the resolution 1Mb in the mouse brain. A) ARI between the true labels and the k‐means predicted labels of multiplexed DNA FISH data imputed with different methods and raw data. Cells with a missing rate not exceeding the specified threshold were selected. The number of repetitions is 4. B) UMAP visualization of multiplexed DNA FISH data imputed with different methods and raw data. The probe missing rate is less than 0.8. C) Boundary insulation scores of multiplexed DNA FISH data imputed with different methods and raw data. The probe missing rate is 0.8. Multiplexed DNA FISH data were merged according to cell types. The boundaries are the insulation score peaks at the transition point between A and B compartments. D) Single‐cell insulation score, heatmap, and compartment eigenvector of multiplexed DNA FISH data imputed with different methods and raw data. The cell name is ‘482′. Chr1: 3.2–195.4 Mb. E) UMAP visualization of OPC/Oligo 1 and OPC/Oligo 2 in DNA FISH imputed by ImputeHiFI mode 1. F) Mean spatial distances of lists of cell‐type‐specific locus pairs among single cells. The top 100 cell‐type‐specific locus pairs are detected from imputed multiplexed DNA FISH data (the result of ImputeHiFI mode 1). G) The proportion of marker genes for oligodendrocyte progenitor and mature oligodendrocyte within the cell type‐specific locus pairs of OPC/Oligo 1 and OPC/Oligo 2. The top 100 marker genes are detected from Tan et al. scRNA‐seq data. The top 100 cell type‐specific locus pairs are detected from imputed multiplexed DNA FISH data (the result of ImputeHiFI mode 1). H) The marker genes expression of Takei et al.’s RNA FISH data in the mouse brain. I) The enrichment scores of mature oligodendrocyte marker genes on the D^2^ plots of OPC/Oligo 1 and OPC/Oligo 2 (the result of ImputeHiFI mode 1). D^2^ plot is DNA density and distance to the nuclear periphery (DisTP) 2D matrix plot. J) Top: Heatmap of Jiang et al. mouse cerebral cortex bulk Hi‐C data. Resolution 40Kb. The position of the Exoc6b gene is chr6: 84618487‐85069513. Bottom: Bar plot of Allan et al. H3K27Ac and ATAC (Allan et al. data) mean count in oligodendrocyte Progenitor cell (rows 1, 3) and mature oligodendrocyte cell (rows 2, 4). Window size 100 bp. H3K27Ac value range: 0–90. ATAC value range: 0–60. K) Spatial distance heatmap of merged OPC/Oligo 1 and OPC/Oligo 2 cells (the result of ImputeHiFI mode 1). L) Spatial distance of locus pairs chr6: (84,86) Mb on imputed multiplexed DNA FISH data (the result of ImputeHiFI mode 1). M) Single‐cell spatial distance heatmap and 3D coordinates of chr6: 73–90 Mb on imputed multiplexed DNA FISH data (the result of ImputeHiFI mode 1).

To demonstrate the effectiveness of ImputeHiFI in enhancing multiplexed DNA FISH data clustering, we applied it to Takei et al.’s 1Mb mouse brain data.^[^ ^23^ ^]^ ImputeHiFI mode 1 utilized Tan et al.’s scHi‐C and scRNA‐seq data.^[^ ^32^ ^]^ ImputeHiFI modes 1 and 2 utilized Takei et al.’s RNA FISH data (Figure 4A,B).^[^ ^23^ ^]^ We observed that distinct cell types were distinguishable after data imputation using ImputeHiFI mode 1 (**Figure** **5**B). The distinction between cell types became less clear in the data imputed using ImputeHiFI mode 2, such as for Ndnf and Pvalb (Figure 5B). In contrast, data imputed using other methods did not display cell types (Figure 5B). Takei et al.^[^ ^23^ ^]^ used RNA FISH data for clustering and employed marker genes to determine the type of each cell cluster. These cell types serve as the ground truth labels. We evaluated the effectiveness of clustering using the Adjusted Rand Index (ARI) and found that ImputeHiFI mode 1 outperformed other methods across various rates of missing data (Figure 5A). Clustering the imputed data by ImputeHiFI mode 1 can increase ARI by 0.3 compared to that of the raw data. In summary, ImputeHiFI enhances the clustering capabilities of multiplexed DNA FISH data in tissues containing complex cell types.

Next, we evaluated the impact of imputation on compartment detection. In the data merged according to cell types, we observed that ImputeHiFI mode 1 significantly improved the insulation values at compartment transition sites (Figure 5C; Figures S8 and S9, Supporting Information), and the overall chromosome structure got clearer. For instance, in Pvalb cells, the raw data displayed significant ambiguity, whereas the imputation using ImputeHiFI clarified the overall chromosomal structure (Figure S8, Supporting Information). Furthermore, we presented the results for compartments and insulation scores in single cells. ImputeHiFI mode 1 preserved the original chromosomal structure during imputation (Figure 5D; Figures S10 and S11, Supporting Information) and achieved higher insulation scores (Figure S10B, Supporting Information). Overall, the multiplexed DNA FISH data imputed by ImputeHiFI exhibited distinct compartment structures.

We demonstrated that the multiplexed DNA FISH data imputed by ImputeHiFI mode 1 can reveal cell subtypes. After imputation, we observed that Opc/Oligo (oligodendrocyte progenitor cells and oligodendrocytes) divided into two clusters (Figure 5E), which we named Opc/Oligo 1 and Opc/Oligo 2, respectively. We identified cell‐type‐specific locus pairs for Opc/Oligo 1 and Opc/Oligo 2 (Figure 5F; Figure S12, Supporting Information) and compared these with the marker genes for oligodendrocyte progenitors and mature oligodendrocytes^[^ ^32^ ^]^ by calculating the overlap ratios. We found that Opc/Oligo 1 is more likely to be oligodendrocyte progenitor cells, and Opc/Oligo 2 is more likely to be mature oligodendrocyte cells (Figure 5G). Further validation was performed using gene expression data from RNA FISH.^[^ ^23^ ^]^ We observed high expression of Olig1 and Olig2 in Opc/Oligo 1, while Plp1 and Mog were highly expressed in Opc/Oligo 2 (Figure 5H). Olig1 and Olig2 are two crucial transcription factors for development and maturation of oligodendrocyte progenitor cells.^[^ ^43^, ^44^, ^45^, ^46^ ^]^ They guide neural stem cells towards the oligodendrocyte lineage during early neural development. The primary function of mature oligodendrocytes is to produce myelin, and Plp1,^[^ ^47^, ^48^ ^]^ as a key structural component of myelin, is highly expressed during this phase. Mature oligodendrocytes may receive signals from neurons that promote the expression of myelin proteins to support the formation and maintenance of myelin, further accelerating the propagation of action potentials and supporting neural signal transmission. The protein encoded by the Mog gene, also known as Myelin Oligodendrocyte Glycoprotein, is a crucial component of central nervous system myelin.^[^ ^49^ ^]^ In mature oligodendrocytes, increased Mog expression contributes significantly to the formation and maintenance of myelin. We then analyzed the positioning of marker genes relative to the nuclear membrane. Previous studies have indicated that genes positioned further from the nuclear membrane within the nucleus tend to exhibit higher expression levels.^[^ ^13^, ^50^, ^51^, ^52^ ^]^ Che et al. designed the D^2^ plot to map DNA density and the distance to the nuclear periphery (DisTP) within the cell nucleus.^[^ ^53^ ^]^ Employing the D^2^ Plot, we noted that the mature oligodendrocyte marker gene is located near the nuclear membrane in Opc/Oligo 2 (Figure 5I), and the oligodendrocyte progenitor marker gene is located near the nuclear membrane in Opc/Oligo 1 (Figure S13A, Supporting Information).

ImputeHiFI mode 1 can reconstruct the interactions between the potential enhancer and promoter. In high‐resolution bulk data of the mouse cerebral cortex,^[^ ^54^ ^]^ the Exoc6b gene is located within a topologically associating domain (TAD), which also interacts with adjacent TAD (Figure 5J). Active regions of genes are associated with high signals of H3K27Ac. ATAC signals are utilized to mark open chromatin regions on the genome. These regions are typically associated with gene regulatory elements such as promoters, enhancers, and other regulatory areas, serving as potential binding sites for transcription factors and other DNA‐binding proteins. In the adjacent TAD region of the Exoc6b gene, we observed higher signals of H3K27Ac and ATAC in mature oligodendrocyte cells (Figure 5J),^[^ ^55^ ^]^ suggesting that this region is more active in mature oligodendrocyte cells and may contain potential enhancer. Correspondingly, Exoc6b exhibits higher expression levels in mature oligodendrocyte cell types in scRNA‐seq data (Figure S14, Supporting Information).^[^ ^32^ ^]^ In the data imputed by ImputeHiFI mode 1, we observed a closer spatial distance between Exoc6b (at the 84Mb locus) and potential enhancer regions (at the 86Mb locus) in OPC/oligo 2 data (Figure 5K,L). A closer spatial distance suggests potential chromosomal interactions between these regions. This difference was also significantly observed at the single‐cell level, with the spatial distance between loci 84 and 86 being relatively closer in single cells from OPC/Oligo 2 (Figure 5M). Summarizing these observations, we can infer that in mature oligodendrocyte cells, the expression of Exoc6b is regulated through the chromatin interactions between its promoter and distal enhancers. The high expression of Exoc6b may facilitate the targeted transport of myelin proteins and lipid vesicles to the plasma membrane, which is crucial for the formation and maintenance of myelin.^[^ ^56^ ^]^

### 2.5 ImputeHiFI Enhances the Detection of Cell‐Type‐Specific Loops

We applied ImputeHiFI to three high‐resolution datasets for imputation and identified chromatin loops from the imputed data. These datasets include the mouse brain 25 kb dataset provided by Takei et al.,^[^ ^23^ ^]^ which contains 60 loci per chromosome covering a 1.5Mb chromosomal region; the mESC 25 kb dataset provided by Takei et al.,^[^ ^22^ ^]^ which contains 60 loci per chromosome across the same 1.5Mb chromosomal region; and the mESC 5 kb dataset provided by Huang et al.,^[^ ^28^ ^]^ which contains 41 loci around the Sox2 locus, spanning 206 kb on chromosome 3. As previously mentioned, all three datasets employed multimodal multiplexed DNA FISH and RNA FISH techniques.

**Figure 6:**
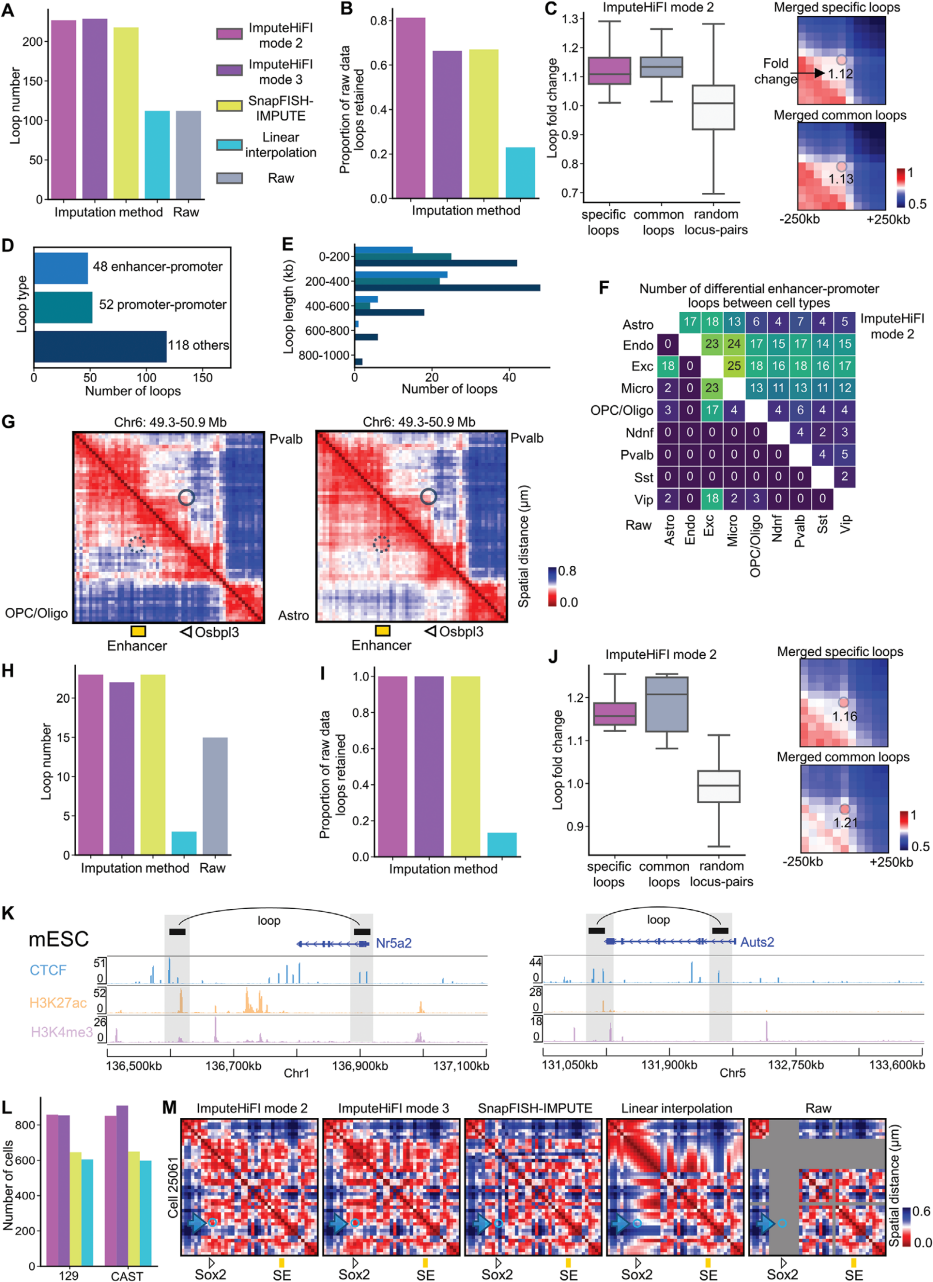
The imputation results of three datasets: (A‐G) Takei et al. multiplexed DNA FISH data at the resolution of 25 kb in mouse brain cells, (H‐K) Takei et al. multiplexed DNA FISH data at the resolution in mESCs, and (L‐M) Huang et al. multiplexed DNA FISH data at the resolution 5 kb in mouse embryonic stem cells. A) Loop number in imputed data and raw data. B) Proportion of the loops from the raw data retained after imputation with different methods. C) Left: Loop fold change is defined as the ratio of the central contacts to the mean of the neighboring contacts (−250 kb to +250 kb). Specific loops refer to those unique in the imputed data by ImputeHiFI mode 2 compared to the raw data. Common loops are those shared between the raw data and the imputed data by ImputeHiFI mode 2. Right: Proximity score matrix heatmap of merged specific loops and common loops. D) Loop numbers for three types of loops in the imputed data by ImputeHiFI mode 2. E) Loop length is the genomic distance between loop endpoints. The Y‐axis represents the loop length interval. ImputeHiFI mode 2 result. F) The number of differential enhancer‐promoter loops between cell types. The upper triangle displays the results of the imputed data by ImputeHiFI mode 2, while the lower triangle shows results from raw data. G) Spatial distance matrix heatmap of the imputed data by ImputeHiFI mode 2. The position of the Osbpl3 gene is chr6: 50293330‐50382837. H) Same as (A). I) Same as (B). J) Same as (C). K) Two enhancer‐promoter loops in the imputed data by ImputeHiFI mode 2. The bottom three tracks are mESC CTCF, mESC H3K27ac, and mESC H3K4me3 ChIP‐seq data. The positions of the Nr5a2 and Auts2 genes are chr1: 136845314‐136960380 and chr5: 131437333‐132543220, respectively. L) Quantification of cells exhibiting Sox2 promoter‐super enhancer interaction on 129 and cast alleles. The Sox2 promoter is located at chr3: 34645000–34655000, and the super‐enhancer is at chr3: 34755000–34765000. Cells with spatial distances shorter than the median between these regions were identified as having a Sox2 promoter‐super enhancer interaction. M) Single‐cell heatmaps of spatial distance matrices from multiplexed DNA FISH data. The cell is named ‘25061′, the allele is ‘129′, and the region covers chr3: 34601078‐34806078. SE means super enhancer.

In the mouse brain 25 kb dataset provided by Takei et al., we observed that more loops were detectable after imputation with ImputeHiFI (**Figure** **6**A). Compared to other methods, ImputeHiFI demonstrated superior performance in preserving loops detected in the raw data (Figure 6B). To investigate the characteristics of these additionally detected loops, we compared the ImputeHiFI mode 2 specific loops, common loops, and randomly selected locus pairs, revealing significant peaks at the central regions for both ImputeHiFI mode 2 specific loops and common loops (Figure 6C). Furthermore, we identified more enhancer‐promoter loops in the imputed data than in the raw data (Figure 6D; Figure S15A, Supporting Information), with the genomic distances of these loops predominantly ranging from 200 to 400 kb (Figure 6E). We also found more differential enhancer‐promoter loops across different cell types in the imputed data (Figure 6F). For example, Pvalb cell types exhibited higher expression levels of Osbpl3 (Figure S16, Supporting Information). Osbpl3 contributes to membrane stability and impacts calcium channel function, maintaining the calcium signaling needed by Pvalb neurons.^[^ ^57^ ^]^ We noted that in Pvalb cell types, the spatial distance between Osbpl3 and potential enhancers was closer than that in OPC/Oligo and Astro cell types (Figure 6G).

In the mESC 25 kb dataset provided by Takei et al., we observed that using ImputeHiFI mode 2, mode 3, and SnapFISH‐IMPUTE for data imputation, effectively preserves the loop in raw data (Figure 6I). ImputeHiFI mode 2 specific loops exhibited significant peaks in the central region (Figure 6J). CTCF contributes to the formation and stabilization of chromatin loops by binding to specific sequences on the chromatin. H3K27Ac is commonly associated with active regions of genes, while H3K4me3 is primarily found in the promoter areas of genes. Using ImputeHiFI mode 2 imputed data, we identified two enhancer‐promoter loops associated with the Nr5a2 and Auts2 genes, respectively (Figure 6K). Nr5a2 positively regulates the expression of key pluripotency maintenance factors, including Oct4, Sox2, and Nanog. These factors form a regulatory network for maintaining the undifferentiated state and high regenerative capacity of stem cells. High expression of Nr5a2 is an integral part of this network, contributing to the maintenance of cellular pluripotency.^[^ ^58^, ^59^ ^]^ Auts2, a protein that plays a crucial role in neural development, is expressed in mESCs. By regulating specific gene expression patterns, Auts2 helps maintain the pluripotency of mESCs and prevents premature differentiation.^[^ ^60^, ^61^, ^62^ ^]^ In summary, the imputed data can retain the raw loops while uncovering additional loops associated with mESCs.

In the mESC 5 kb dataset provided by Huang et al., Sox2 forms a loop with its corresponding super‐enhancer. However, at the single‐cell level, many cells exhibit data missing in this specific interaction (Figure S17D, Supporting Information). After imputation with ImputeHiFI, the loop between Sox2 and the super‐enhancer is restored in more single cells (Figure 6L). For instance, the raw data failed to detect the Sox2 and super‐enhancer loci, and ImputeHiFI successfully restored the loop in cell ‘25061′ (Figure 6M).

## 3 Discussion

Unlike existing methods such as SnapFISH‐IMPUTE, ImputeHiFI is the first to leverage RNA FISH and scHi‐C data. We discovered that scHi‐C data contains abundant information on cell types and is highly consistent with multiplexed DNA FISH data. The increasing maturity of multimodal imaging and sequencing technologies provides broader applications for ImputeHiFI. For instance, the MUSIC (multinucleic acid interaction mapping in single cells) technique^[^ ^63^ ^]^ allows for the simultaneous analysis of multiple chromatin interactions, gene expression, and RNA‐chromatin associations within a single cell nucleus. Multimodal data offer the structural and functional dynamics of single‐cell genomes. Currently, integration methods are required to pair scHi‐C with multiplexed DNA FISH data, making the pairing results dependent on the integration methods. As multimodal scHi‐C and multiplexed DNA FISH technologies advance, using scHi‐C and multiplexed DNA FISH data from the same single cell will enhance the accuracy of the imputation results. Moreover, ImputeHiFI relies on structurally similar chromosomes for imputation. When the missing rate is too high, it affects the computation of similarity and, consequently, the effectiveness of ImputeHiFI. Looking ahead, existing experimental techniques can simultaneously measure interaction and methylation data of chromosomal segments^[^ ^35^, ^37^ ^]^ or measure the spatial positioning of chromosomes and histone modifications using imaging methods.^[^ ^21^, ^22^, ^23^ ^]^ The epigenetic changes significantly impact chromosomal structure, and computational methods have been developed to infer Hi‐C data using such multimodal data.^[^ ^64^ ^]^ In the future, incorporating additional modalities could be considered to assist in inferring missing DNA locations.

ImputeHiFI can be applied to various imaging technologies, such as DNA MERFISH and DNA seqFISH+, and at different resolutions, including 5 kb, 25 kb, and 1 Mb. For instance, when processing the 1 Mb DNA seqFISH+ data of the mouse brain,^[^ ^23^ ^]^ ImputeHiFI significantly enhances the clustering of DNA seqFISH+ data, allowing for accurate differentiation between cell subtypes such as oligodendrocyte progenitors and mature oligodendrocytes. For analysis of 25 kb DNA seqFISH+ data from the mouse brain,^[^ ^23^ ^]^ ImputeHiFI effectively preserves known chromatin loops and uncovers loops not detected in the raw data. For example, ImputeHiFI identifies the enhancer and promoter loop in Pvalb cells, potentially linked to the Osbpl3 gene's role in regulating calcium signaling. When processing the 25 kb DNA seqFISH+ data of mESC,^[^ ^22^ ^]^ new chromatin loops associated with key pluripotency maintenance factors were discovered. When processing the 5 kb DNA MERFISH data of mESC,^[^ ^28^ ^]^ the chromatin loop of Sox2 and super‐enhancer was accurately recovered in more single cells.

## 4 Conclusions

The rapid development of multimodal imaging and sequencing technologies has provided a robust groundwork for imputing multiplexed DNA FISH data. ImputeHiFI enhances the completeness of the data and, more importantly, improves the depth and accuracy of downstream analyses, including clustering analysis, compartment detection, cell subtype identification, and chromatin loop recognition. The imputed data better reveal the gene regulatory mechanisms and variations in chromatin structures across different cell types, which are essential for interpreting the 3D genome functions within complex tissues. Boninsegna et al. integrated Hi‐C data, chromatin‐lamina interactions, and multi‐directional chromatin contacts to construct populations of single‐cell genome architectures.^[^ ^65^ ^]^ They predicted the nuclear localization of genes and nucleosomes, local chromatin compaction, and the spatial segregation of functionally related chromatin. Tan et al. introduced the C.Origami method, which leverages DNA sequences and two cell type‐specific genomic features, i.e., CTCF binding and chromatin accessibility, to predict new cell type‐specific chromatin organizations.^[^ ^64^ ^]^ However, ImputeHiFI does not utilize chromatin‐lamina interactions, multi‐way chromatin contacts, CTCF binding, and chromatin accessibility, limiting its capability to explore complex chromosomal states within the nucleus. We will continue to develop ImputeHiFI to leverage more multimodal information.

## 5 Experimental Section

### Data Processing of Multiplexed DNA FISH Data

In the dataset of Huang et al.’s 5 kb multiplexed DNA FISH data, cells exhibiting a missing probe rate of more than 80% were excluded. In the dataset from Takei et al.’s 25 kb and 1 Mb mouse brain and mESCs, each chromosome contained two homologous chromosomes in a single cell. One homologous chromosome typically had a lower missing rate, averaging 60%, and the other had a higher missing rate, with an average of 90% (Figure S18, Supporting Information). The homologous chromosome with the lower missing rate from each chromosome was chosen and kept homologous chromosomes with probe missing rates not exceeding 80% for subsequent processing. One chromosome of the multiplexed DNA FISH data was represented as *X*, an *N*  × 3 matrix, where *N* is the number of loci on a chromosome, and each row of *X* represents a locus with its 3D coordinates (*x*, *y*, *z*). For example,*X_i_* = (*x_i_*,*y_i_*,*z_i_*) denotes the 3D coordinate of the *i*‐th locus.

### Constructing the Pairwise Spatial Distance of Multiplexed DNA FISH Data

The physical distance between two specific DNA segments (known as loci) in micrometers (µm)was measured. This distance represented the straight‐line distance between two points in a 3D space. For example, the distance between two loci, *L_i_* and *L_j_*, with coordinates (*x_i_*,*y_i_*,*z_i_*) and (*x_j_*,*y_j_*,*z_j_*), was calculated using the formula:

(1) $$D_{ij}=\sqrt{{\left(x_{i}-x_{j}\right)}^{2}+{\left(y_{i}-y_{j}\right)}^{2}+{\left(z_{i}-z_{j}\right)}^{2}}$$

where *D_ij_* is the Euclidean distance between *L_i_* and *L_j_*. If either or both loci in a pair were not fully detected, their distance was marked as “not applicable” (NA) in the dataset.

### Proximity Measurements Between Genomic Loci

Following the method described by Takei et al.,^[^ ^23^ ^]^ the distance matrix was converted into the proximity score matrix. Gaussian kernel was employed to transform spatial distances into a standardized range from 0 to 1. This standardization assigned a proximity score to each pair of loci based on their normalized spatial distance. The proximity score, *K*_*i*,*j*_, for loci *i* and *j*, was calculated using the *D_ij_*. The formula for this calculation was as follows:

(2) $$K_{i,j}=\exp\left(-\frac{D_{i,j}^{2}}{\sigma^{2}}\right)$$

where σ is the bandwidth parameter controlling the rate at which the proximity score decreases with increasing spatial distance. The 50th percentile of the spatial distance matrix was employed as the value for σ. For instance,σ was set as 1.7 for the 1 Mb multiplexed DNA FISH data (chromosome 1, the dataset of Takei et al.) of excitatory neurons, where a spatial distance of 1.7 µm between two loci yielded a 0.5 proximity score. As the distances increased to 3.4 µm, the proximity scores reached zero. The spatial distance matrices and corresponding proximity score matrices of different cell types are shown in Figures S19 and S20, Supporting Information.

### Structure Similarity Metrics—Root Mean Square Deviation

To compare the 3D structures of two chromosomes, the Root Mean Square Deviation (RMSD) was used as a metric. This metric was based on the spatial coordinates of the structures. The formula for RMSD is:

(3) $$\mathrm{RMSD}=\sqrt{\frac{1}{n}\sum_{i=1}^{n}{\left(L_{i}^{A}-L_{i}^{B^{*}}\right)}^{2}}$$

where *L^A^* and *L^B^* represent the coordinates of the two chromosome structures *A* and *B*, and *n* is the number of loci. $L^{B^{*}}$ is obtained by translating and rotating *L^B^*. To align *L^B^* with *L^A^*, the difference in the centroid positions of *L^A^* and *L^B^* were calculated. The centroid of a set of points was the arithmetic mean position of all the points. The centroid of *L^A^*, denoted by *C_A_*, is calculated as:

(4) $$C_{A}=\frac{1}{n}\sum_{i=1}^{n}L_{i}^{A}$$

and the centroid of *L^B^*, denoted by *C_B_*, is calculated as:

(5) $$C_{B}=\frac{1}{n}\sum_{i=1}^{n}L_{i}^{B}$$

The translation vector *t*, which represented the difference between the centroids of *L^A^* and *L^B^*, was then given by: *t* = *C_A_* − *C_B_*. This translation vector *t* was used to shift the centroid of *L^B^* to the centroid of *L^A^*.

Next, the orthogonal Procrustes problem was solved to find the optimal rotation matrix *R* that aligns *L^A^* with *L^B^*. This problem was expressed as:

(6) $$\min_{R}|L^{A}-L^{B}R-t{|}_{F}$$

where the Frobenius norm | · |_*F*_ was a measure of the Euclidean distance between *L^A^* and the transformed *L^B^*. After obtaining the optimal rotation matrix *R*, the transformation was applied to get $L^{B^{*}}$, which was the best alignment of *L^B^* with *L^A^*. This was given by: $L^{B^{*}}=L^{B}R+t$.

Finally, RMSD between *L^A^* and $L^{B^{*}}$ was computed to measure the similarity between the two chromosome structures.

### Structure Similarity Metrics—Mean Square Error

Beyond RMSD, the Mean Square Error (MSE) between the proximity scores of chromosome structures *A* and *B* was also determined. The MSE was given by:

(7) $$\mathrm{MSE}=\frac{1}{n\left(n-1\right)/2}\sum_{i=1}^{n}\sum_{j=i+1}^{n}{\left(K_{i,j}^{A}-K_{i,j}^{B}\right)}^{2}$$

where *n* represents the total number of locus pairs, *K^A^* and *K^B^* are the proximity score matrixes of chromosome structures *A* and *B*. Lower values for RMSD and MSE suggest a higher similarity between chromosome structures *A* and *B*.

### Algorithm of ImputeHiFI

ImputeHiFI had three modes to accommodate different data scenarios (Table 1).

### ImputeHiFI Mode 1

ImputeHiFI mode 1 required multimodal multiplexed DNA FISH and RNA FISH data, multi‐omics scHi‐C, and scRNA‐seq data. Multiplexed DNA FISH was referred to as DNA FISH (Figure 3).

Step 1: Prepared neighbor DNA FISH and scHi‐C data.

First, DNA FISH data *X*, an *N*  × 3 matrix was transformed, into a proximity score matrix *K*, an *N*  × *N* matrix, denoted as *K*_FISH_. Within *K*_FISH_, the entries that contained values were referred to as observed locus pairs, while those without values were termed missing locus pairs.

After pre‐clustering using RNA FISH data, the similarity between chromosomal structures within the same cell type was calculated using RMSD and constructed a neighbor graph. Subsequently, by computing the average of *m* neighbors, the neighbor DNA FISH data was merged, denoted as *K*_neighbor‐FISH_.

The scHi‐C data was represented for a single chromosome as *H*, an *N*  × *N* matrix, where *N* was the number of loci on the chromosome, and each entry *H*_*i*,*j*_ of the matrix represents the contact frequency between loci *i* and *j*. MaxFuse was utilized to pair RNA FISH data with scRNA‐seq data. MaxFuse was a cross‐modal integration method that effectively integrates features from different data modalities with weak feature links through iterative co‐embedding, data smoothing, and cell matching steps.^[^ ^40^ ^]^ RNA FISH data typically measured no more than 300 genes, whereas scRNA‐seq data could measure up to 20 000 genes. MaxFuse could effectively integrate these two modalities. scGAD was employed to pair scRNA‐seq data with scHi‐C data. scGAD calculated gene‐associated domain values at the scHi‐C data and used Seurat to integrate scHi‐C with scRNA‐seq data.^[^ ^41^ ^]^ Based on the integration results, the nearest cells were identified to construct pairings between RNA FISH and scRNA‐seq cells, scRNA‐seq and scHi‐C cells, and then obtained DNA FISH and scHi‐C cellular pairs. Subsequently, a neighbor graph based on the low‐dimensional representation of scHi‐C was built and computed the average of *m* neighbors to obtain the neighbor scHi‐C data denoted as *H*_neighbor‐scHi − C_. MaxFuse had shown superior performance when integrating multi‐omics datasets with weakly linked features. scGAD was the only method capable of accurately integrating scHi‐C and scRNA‐seq data. Neighbor DNA FISH data was merged to mitigate the impact of missing loci on subsequent imputation. Neighbor scHi‐C data was merged to mitigate the impact of sparsity on subsequent imputation. The merged data *K*_neighbor‐FISH_ and *H*_neighbor‐scHi − C_ were passed to step two.

Step 2: Imputed proximity score matrix of DNA FISH data by borrowing information from neighbor data.

The imputation process was formalized as a constrained optimization problem. In this problem, non‐negative weights *a* and *b* were sought that minimize the discrepancy between the observed DNA FISH data and a linear combination of the scHi‐C data and the neighboring DNA FISH data. The scHi‐C data exhibited interaction strengths that decayed with increasing genomic distance, and similarly, the DNA FISH data also displayed spatial distances that expanded with greater genomic distances. This optimization was performed on each genomic distance *v*. Additionally, it was found that the effective interactions in scHi‐C data were primarily concentrated within genomic distances of <30 (Figure S21, Supporting Information). The genomic distance was calculated, denoted as *k*, which maximized the Spearman correlation between single‐cell Hi‐C and DNA FISH data (Figure S22, Supporting Information). When *v* was less than or equal to *k*, the Hi‐C weight bound *w*_bound_ was set to 0.4; when *v* was greater than *k*, the Hi‐C weight bound *w*_bound_ was set to 0. The sum values of locus pairs in the genomic distance *v* for *K*_FISH_, *K*_neighbor‐FISH_, and *H*_neighbor‐scHi − C_were calculated. The sum values of observed locus pairs in genomic distance *v* were represented as follows:

(8) $$S_{\mathrm{FISH}}\left(\mathrm{observed}\right)=\sum_{j-i=v}K_{\mathrm{FISH}}\left(i,j\right)$$

(9) $$S_{\mathrm{neighbor}-\mathrm{FISH}}\left(\mathrm{observed}\right)=\sum_{j-i=v}K_{\mathrm{neighbor}-\mathrm{FISH}}\left(i,j\right)$$

(10) $$S_{\mathrm{neighbor}-\mathrm{scHi}-C}\left(\mathrm{observed}\right)=\sum_{j-i=v}H_{\mathrm{neighbor}-\mathrm{scHi}-C}\left(i,j\right)$$

where (*i*, *j*) ∈ observedlocuspairs. The sum values of missing locus pairs in genomics distance *v* were represented as follows:

(11) $$S_{\mathrm{neighbor}-\mathrm{FISH}}\left(\mathrm{missing}\right)=\sum_{j-i=v}K_{\mathrm{neighbor}-\mathrm{FISH}}\left(i,j\right)$$

(12) $$S_{\mathrm{neighbor}-\mathrm{scHi}-C}\left(\mathrm{missing}\right)=\sum_{j-i=v}H_{\mathrm{neighbor}-\mathrm{scHi}-C}\left(i,j\right)$$

where (*i*, *j*) ∈ missinglocipairs. Then the original matrices *K*_FISH_, *K*_neighbor‐FISH_, and *H*_neighbor‐scHi − C_ were transformed into normalized *P* matrices in genomic distance *v*. This involved normalizing the observed and missing locus pairs so that their sums equaled one. The normalization formulas were as follows:

(13) $$P_{\mathrm{FISH}}\left(i,j\right)=\frac{K_{\mathrm{FISH}}\left(i,j\right)}{S_{\mathrm{FISH}}\left(\mathrm{observed}\right)}$$

(14) $$P_{\mathrm{neighbor}-\mathrm{FISH}}\left(i,j\right)=\left\{\begin{array}{cc} \frac{K_{\mathrm{neighbor}-\mathrm{FISH}}\left(i,j\right)}{S_{\mathrm{neighbor}-\mathrm{FISH}}\left(\mathrm{observed}\right)}, & \mathrm{if}\left(i,j\right)\in \mathrm{observed}\mathrm{loci}\mathrm{pairs}\\ \frac{K_{\mathrm{neighbor}-\mathrm{FISH}}\left(i,j\right)}{S_{\mathrm{neighbor}-\mathrm{FISH}}\left(\mathrm{missing}\right)}, & \mathrm{if}\left(i,j\right)\in \mathrm{missing}\mathrm{loci}\mathrm{pairs} \end{array}\right.$$

(15) $$P_{\mathrm{neighbor}-\mathrm{scHi}-C}\left(i,j\right)=\left\{\begin{array}{cc} \frac{K_{\mathrm{neighbor}-\mathrm{scHi}-C}\left(i,j\right)}{S_{\mathrm{neighbor}-\mathrm{scHi}-C}\left(\mathrm{observed}\right)}, & \mathrm{if}\left(i,j\right)\in \mathrm{observed}\mathrm{loci}\mathrm{pairs}\\ \frac{K_{\mathrm{neighbor}-\mathrm{scHi}-C}\left(i,j\right)}{S_{\mathrm{neighbor}-\mathrm{scHi}-C}\left(\mathrm{missing}\right)}, & \mathrm{if}\left(i,j\right)\in \mathrm{missing}\mathrm{loci}\mathrm{pairs} \end{array}\right.$$

where *j* − *i* = *v*. The optimization problem can be expressed as follows:

(16) $$\begin{array}{ccc} & & \min_{a,b}\sum_{(i,j)\in \mathrm{observed}\mathrm{loci}\mathrm{pairs}}\left(a\cdot P_{\mathrm{neighbor}-\mathrm{scHi}-C}\left(i,j\right)\right.\\ & & {\left.\;\;\;\;\;+b\cdot P_{\mathrm{neighbor}-\mathrm{FISH}}\left(i,j\right)-P_{\mathrm{FISH}}\left(i,j\right)\right)}^{2}\\ & & s.t.a\geq w_{\mathrm{bound}},b\geq 0,a+b=1 \end{array}$$

where *j* − *i* = *v*. The non‐negativity constraints on parameters *a* and *b* prevent non‐physical solutions, avoiding negative contributions from the underlying data. The SLSQP (Sequential Least Squares Programming)^[^ ^66^ ^]^ method from SciPy^[^ ^67^ ^]^ was employed to solve the optimization function, which allowed it to handle equality and inequality constraints efficiently.

Upon solving this constrained optimization problem, the missing locus pairs in *P*_FISH_ are imputed using the optimal weights *a* and *b* as follows:

(17) $$P_{\mathrm{imputed}-\mathrm{FISH}}\left(i,j\right)=a\cdot P_{\mathrm{neighbor}-\mathrm{scHi}-C}\left(i,j\right)+b\cdot P_{\mathrm{neighbor}-\mathrm{FISH}}\left(i,j\right)$$

where (*i*, *j*) ∈ missinglocipairs. The sum values of missing locus pairs in the genomic distance *v* for *P*_FISH_ is calculated as follows:

(18) $$\begin{array}{ccc} & & \mathrm{proportion}\;\mathrm{of}\;\mathrm{observed}\;\mathrm{values}=\\ & & a\cdot \frac{S_{\mathrm{neighbor}-\mathrm{FISH}}\left(\mathrm{observed}\right)}{S_{\mathrm{neighbor}-\mathrm{FISH}}\left(\mathrm{observed}\right)+S_{\mathrm{neighbor}-\mathrm{FISH}}\left(\mathrm{missing}\right)}\\ & & +b\cdot \frac{S_{\mathrm{neighbor}-\mathrm{scHi}-C}\left(\mathrm{observed}\right)}{S_{\mathrm{neighbor}-\mathrm{scHi}-C}\left(\mathrm{observed}\right)+S_{\mathrm{neighbor}-\mathrm{scHi}-C}\left(\mathrm{missing}\right)} \end{array}$$

(19) $$\begin{array}{ccc} S_{\mathrm{FISH}}\left(\mathrm{imputed}\right) & = & \frac{S_{\mathrm{FISH}}\left(\mathrm{observed}\right)}{\mathrm{proportion}\mathrm{of}\mathrm{observed}\mathrm{values}}\\ & & -S_{\mathrm{FISH}}\left(\mathrm{observed}\right) \end{array}$$

Finally, *K*_imputed‐FISH_ was calculated as follows:

(20) $$K_{\mathrm{imputed}-\mathrm{FISH}}\left(i,j\right)=\left\{\begin{array}{cc} P_{\mathrm{imputed}-\mathrm{FISH}}\left(i,j\right)\cdot S_{\mathrm{FISH}}\left(\mathrm{imputed}\right), & \mathrm{if}\left(i,j\right)\in \mathrm{missing}\mathrm{loci}\mathrm{pairs}\\ K_{\mathrm{FISH}}\left(i,j\right), & \mathrm{if}\left(i,j\right)\in \mathrm{observed}\mathrm{loci}\mathrm{pairs} \end{array}\right.$$

Step 3: Infer 3D coordinates of undetected loci.

Following the approach of Varoquaux et al.,^[^ ^42^ ^]^ Poisson distribution was employed to describe the relationship between the 3D coordinates of loci and the proximity scores of locus pairs. In Hi‐C experiments, the contact frequency between genomic loci could be considered independent random events, where the frequency of occurrence was associated with their physical distance. The Poisson distribution supported the modeling assumption that the contact frequency was inversely proportional to the physical distance, meaning that greater distances corresponded to lower contact frequencies. Contact frequency was positively correlated with the proximity score. The contact frequency was obtained by scaling and rounding the proximity score. Here, the 3D coordinates of loci were treated as the Poisson parameter λ, while the proximity score was treated as the Poisson random variable *k*. For undetected loci, the inference of their 3D coordinates was formulated as maximizing the Poisson likelihood function problem.

To simplify the following equation, two functions: *f_D_* (*X*_*i*,*j*_) and *f_K_* (*D*_*i*,*j*_)were introduced. The function *f_D_* (*X*_*i*,*j*_) calculated the Euclidean distance between two points, *X_i_* and *X_j_*. Their coordinates were (*x_i_*,*y_i_*,*z_i_*) and (*x_j_*,*y_j_*,*z_j_*), respectively. The definition was:

(21) $$f_{D}\left(X_{i,j}\right)=\sqrt{{\left(x_{i}-x_{j}\right)}^{2}+{\left(y_{i}-y_{j}\right)}^{2}+{\left(z_{i}-z_{j}\right)}^{2}}$$

The function *f_K_* (*D*_*i*,*j*_) computed the proximity score, *K*_*i*,*j*_, using the distance *D*_*i*,*j*_. The definition was:

(22) $$f_{K}\left(D_{i,j}\right)=\exp\left(-\frac{D_{i,j}^{2}}{\sigma^{2}}\right)$$

The likelihood function could then be expressed as:

(23) $$L\left(X\right)=\prod_{i,j}{\left(f_{K}\left(f_{D}\left(X_{i,j}\right)\right)\right)}^{K_{\mathrm{imputed}-\mathrm{FISH}}\left(i,j\right)}\frac{e^{-f_{K}\left(f_{D}\left(X_{i,j}\right)\right)}}{K_{\mathrm{imputed}-\mathrm{FISH}}\left(i,j\right)!}$$

Taking the logarithm of the likelihood function simplified the expression. The log‐likelihood function was as follows:

(24) $$\begin{array}{ccc} \log L\left(X\right) & = & \sum_{i,j}[K_{\mathrm{imputed}-\mathrm{FISH}}\left(i,j\right)\log\left(f_{K}\left(f_{D}\left(X_{i,j}\right)\right)\right)\\ & & -f_{K}\left(f_{D}\left(X_{i,j}\right)\right)-\log\left(K_{\mathrm{imputed}-\mathrm{FISH}}\left(i,j\right)!\right)] \end{array}$$

By maximizing the log‐likelihood, an optimization function could be expressed as follows:

(25) $$\begin{array}{ccc} \max_{X}\log L\left(X\right) & = & \max_{X}\sum_{i,j}[K_{\mathrm{imputed}-\mathrm{FISH}}\left(i,j\right)\log\left(f_{K}\left(f_{D}\left(X_{i,j}\right)\right)\right)\\ & & -f_{K}\left(f_{D}\left(X_{i,j}\right)\right)] \end{array}$$

where *i*, *j* ∈ Undetectedloci. The objective was to determine the 3D coordinates of these undetected loci that maximize the log‐likelihood function. To achieve this, the L‐BFGS‐B (Limited‐memory Broyden‐Fletcher‐Goldfarb‐Shanno with Bounds)^[^ ^68^ ^]^ algorithm from SciPy^[^ ^67^ ^]^was utilized to optimize and obtain the imputed values for *X*.

### ImputeHiFI Mode 2

ImputeHiFI mode 2 required multimodal multiplexed DNA FISH and RNA FISH data (Figure S5, Supporting Information).

Step 1: Prepare neighbor DNA FISH data.

After initially pre‐clustering based on RNA FISH data, the similarity between chromosomal structures within the same cell type was assessed using RMSD to construct a neighbor graph. The average from *m* neighbors was computed and merged with this data to form the neighbor DNA FISH data, which was denoted as *K*_neighbor‐FISH_. This process was identical to that used in ImputeHiFI mode 1.

Step 2: Imputed proximity score matrix of DNA FISH data by borrowing information from neighbor data.

In ImputeHiFI mode 2, only the neighboring DNA FISH data information was utilized, therefore the objective function and constraints were modified as follows:

(26)

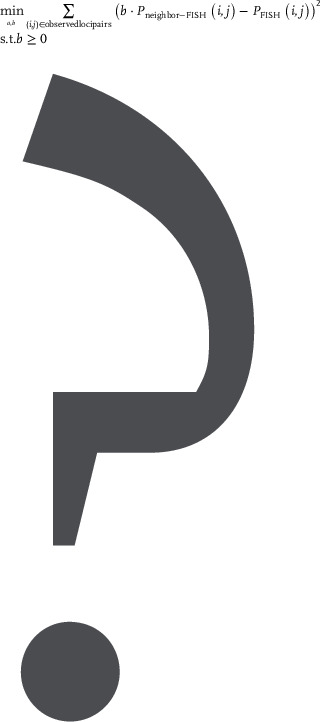




The missing locus pairs in *P*_FISH_ were imputed using the optimal *b* as follows:

(27) $$P_{\mathrm{imputed}-\mathrm{FISH}}\left(i,j\right)=b\cdot P_{\mathrm{neighbor}-\mathrm{FISH}}\left(i,j\right)$$

The sum values of missing locus pairs in genomic distance *v* for *P*_FISH_ was calculated as follows:

(28) $$\begin{array}{ccc} & & \mathrm{proportion}\mathrm{of}\mathrm{observed}\mathrm{values}=\\ & & \frac{S_{\mathrm{neighbor}-\mathrm{scHi}-C}\left(\mathrm{observed}\right)}{S_{\mathrm{neighbor}-\mathrm{scHi}-C}\left(\mathrm{observed}\right)+S_{\mathrm{neighbor}-\mathrm{scHi}-C}\left(\mathrm{missing}\right)} \end{array}$$

(29) $$S_{\mathrm{FISH}}\left(\mathrm{imputed}\right)=\frac{S_{\mathrm{FISH}}\left(\mathrm{observed}\right)}{\mathrm{proportion}\mathrm{of}\mathrm{observed}\mathrm{values}}-S_{\mathrm{FISH}}\left(\mathrm{observed}\right)$$

Other settings of ImputeHiFI mode 2 remained consistent with mode 1.

Step 3: Inferred 3D coordinates of undetected loci.

Here, all settings of ImputeHiFI mode 2 remained consistent with mode 1.

### ImputeHiFI Mode 3

ImputeHiFI mode 3 only required multiplexed DNA FISH data (Figure S6, Supporting Information).

Step 1: Prepared neighbor DNA FISH data.

RMSD was utilized to evaluate the similarity between chromosomal structures using DNA FISH data. Following this, a neighbor graph was constructed, and calculated the average of *m* neighbors to get *K*_neighbor‐FISH_.

Step 2: Imputed proximity score matrix of DNA FISH data by borrowing information from neighbor data.

Here, all settings of ImputeHiFI mode 3 remained consistent with mode 2.

Step 3: Inferrd 3D coordinates of undetected loci.

Here, all settings of ImputeHiFI mode 3 remained consistent with mode 2.

### Generation of Simulated Multiplexed DNA FISH Data

Simulated data was created using Takei et al.’s 1 Mb multiplexed DNA FISH data in the mouse brain.^[^ ^23^ ^]^ Initially, 446 chromosomes with less than a 30% probe missing rate were selected. Subsequently, the loci of detected probes were used to create the simulated datasets. Specifically, for the valued loci of each chromosome, some loci were randomly masked to achieve the simulated probe missing rate at 50%, 60%, 70%, and 80%.

### Parameter Selection

The impact of the Hi‐C weight bound, the number of neighboring multiplexed DNA FISH cells, and the number of neighboring scHi‐C cells on RMSD, MSE, and ARI (Figure S23, Supporting Information) were evaluated. In ImputeHiFI mode 1, it was observed that when the Hi‐C weight bound exceeded 0.4, it had little effect on ARI, but continued to increase the impact on MSE and RMSD. The default Hi‐C weight bound was set at 0.4. When the number of neighboring scHi‐C cells ranged from 5 to 20, there was no significant effect on ARI; however, ARI slightly declined when this number exceeded 20. The number of neighboring scHi‐C cells did not significantly affect MSE and RMSD. The default number was set at 10. When the number of neighboring multiplexed DNA FISH cells reached 100, the lowest values of RMSD and MSE were observed; exceeding this number, the values of these metrics continued to increase. The default number of neighboring multiplexed DNA FISH cells was set at 100. In ImputeHiFI modes 2 and 3, when the number of multiplexed DNA FISH cells was 100, the lowest values of RMSD and MSE were observed. Exceeding this number, the values of these metrics continued to increase. The number of multiplexed DNA FISH cells had no significant impact on ARI, and the default number of neighboring multiplexed DNA FISH cells was also set at 100.

### Time Cost of ImputeHiFI

ImputeHiFi was implemented in Python and supports multicore processing. When utilizing 20 processing cores, ImputeHiFi could impute data for 1386 cells from the Huang et al. dataset at a 5 kb resolution within 10 min. The imputation time scales approximately linearly with the number of cells.

### Clustering Methods

Decay calculated the ratios of contacts in different genomic distances to the total contacts as the features of each cell.^[^ ^8^ ^]^ Nagano et al. showed that Decay can effectively separate single‐cell Hi‐C data from different cell cycles. scHiCluster used linear convolution and random walk for imputation and then used PCA to get the low‐dimensional representation of scHi‐C data. PCC identified highly variable locus pairs in the frequency interaction map of all cells as the profile of scHi‐C data.^[^ ^38^ ^]^ For the scRNA‐seq data, Scanpy^[^ ^69^ ^]^ was employed for preprocessing and obtained a low‐dimensional representation by applying Principal Component Analysis (PCA) with the dimensionality set to 30. The multiplexed DNA FISH data was transformed into a proximity score matrix and applied PCA to achieve a low‐dimensional representation with the dimensionality set to 30.

### Insulation Score

The approach followed was described by Su et al. to calculate the insulation score in imaging data.^[^ ^21^ ^]^ For a given proximity score matrix, it was first partitioned into windows of fixed size. Subsequently, the average proximity scores within each window (intra value) and the average proximity scores between adjacent windows (inter value) were calculated. The insulation score for each window was defined as $\mathrm{insulation}\mathrm{score}=\frac{\mathrm{intra}\mathrm{value}-\mathrm{inter}\mathrm{value}}{\mathrm{intra}\mathrm{value}+\mathrm{inter}\mathrm{value}}$. The insulation score ranged from 0 to 1. Two highly separated domains would have an insulation score close to 1.

### Compartment Identification

To identify compartments from multiplexed DNA FISH data, a normalized proximity score matrix was generated by dividing the observed proximity score matrix over the expected proximity scores for the genomic distances of the respective locus pairs. The expected proximity scores were obtained by fitting a logarithmic scale linear regression to the locus pair data against genomic distances. Subsequently, a Pearson correlation matrix was computed from the normalized proximity score matrix and conducted a principal component analysis. Based on the positive or negative values of the first principal component (PC1), the corresponding genomic regions were classified into two distinct compartments.^[^ ^21^ ^]^

### Loop Detection

The Single‐Nucleus Analysis Pipeline was employed for multiplexed DNA FISH (SnapFISH) to identify chromatin loops from multiplexed DNA FISH data.^[^ ^70^ ^]^ SnapFISH captured 3D coordinates of genomic segments targeted by FISH and calculates pairwise Euclidean distances. For segment pairs within a genomic distance of 100 Kb to 1 Mb, SnapFISH employed a two‐sample T‐test to compare the observed distances with those in their local genomic neighborhood to get candidate loops. The P‐values were adjusted using a false discovery rate (FDR) control (FDR cutoff 0.1). SnapFISH groups nearby loop candidates into clusters. The pair with the lowest false discovery rate (FDR) was identified and designated as the cluster summit within each cluster.

### D^2^ Plot of DNA Density and Distance to the Nuclear Periphery

DNA density (hereafter referred to as density) and distance to the nuclear periphery (DisTP) were two principal physical characteristics associated with transcriptional activity in the spatial genome organization. The D^2^ algorithm calculated density and DisTP at a high resolution across the whole genome.^[^ ^53^ ^]^ The input for this algorithm consisted of a set of DNA particles. Each particle possessed its spatial coordinates and genomic location, linking the spatial structure with the genomic sequence. The D^2^ algorithm introduced a 3D grid partitioning that assignEd the input DNA particles to different cubes. By adjusting the length of the cubes, the D^2^ algorithm ensured that the number of particles within each cube was roughly equal across different cells, thus allowing for intercellular comparisons. Density was defined as the number of particles within a cube and was smoothed to reduce the impact of noise. The D^2^ algorithm first identified the boundary cubes and then computed the distance from each cube to the nearest boundary cube as the DisTP.

### Data Availability Statement

In this study, scHi‐C (1954 cells) and scRNA‐seq (3517 cells) data was utilized from 14 cell types of the human prefrontal cortex.^[^ ^32^ ^]^ The processed dataset was publicly available at the Gene Expression Omnibus (GEO) under accession number GSE162511. The Takei et al. dataset, which included 2762 cells from 9 cell types of the mouse cerebral cortex, was available at Zenodo with the following https://doi.org/10.5281/zenodo.5507507. 1 Mb dataset included 2460 loci. 25 kb dataset includes 1200 loci. The Takei et al. dataset for mESCs, which included 446 cells from two biological replicates, was available at Zenodo with the following https://doi.org/10.5281/zenodo.3735329.^[^ ^22^ ^]^ 1Mb dataset includes 2460 loci. 25 kb dataset included 1200 loci. The Huang et al. dataset for mESCs, which included 1386 cells, was available at the 4DN data portal with ID 4DNFI9KE6AII. 5 kb dataset included 41 loci.^[^ ^28^ ^]^ The Su et al. study included chromosome imaging data at resolutions of 50 kb (650 loci, 12100 copies of chr21 from the two replicates), 250 kb (935 loci, 3000 copies of chr2), and 3 Mb (1041 loci, 5400 cells of whole genome from 5 biological replicates) for the human IMR90 cell line. The processed data could be accessed at Zenodo at https://doi.org/10.5281/zenodo.3928890.^[^ ^21^ ^]^ The Payne et al. dataset included chromosome imaging data at 2.5 Mb and 10 Mb resolutions for 106 human fibroblast cells and 57 early mouse embryo cells. The processed data was available at Zenodo at https://doi.org/10.5281/zenodo.4299227 ^.[^ ^20^ ^]^ Jiang et al.’s bulk Hi‐C of the mouse brain was available at GEO with access number GSE99363.^[^ ^54^ ^]^ mESC H3K4me3 ChIP‐seq data was available at GEO with access number GSE119663.^[^ ^71^ ^]^ mESC H3K27ac ChIP‐seq data was available at ENCODE with the following DOI:10.17989/ENCSR000CGQ. mESC CTCF ChIP‐seq data was available at ENCODE with the following DOI: 10.17989/ENCSR000CCB. Oligodendrocyte progenitor cells and mature oligodendrocyte cells H3K27ac ChIP‐seq data was available at GEO with access number GSE182245.^[^ ^55^ ^]^ Oligodendrocyte progenitor cells and mature oligodendrocyte cells ATAC‐seq data was available at GEO with access number GSE182558.^[^ ^55^ ^]^ The code of ImputeHiFI was open‐source and publicly available on GitHub at https://github.com/zhanglabtools/ImputeHiFI.

## Conflict of Interest

The authors declare no conflict of interest.

## Supporting information



Supporting Information

## Data Availability

The data that support the findings of this study are available in the supplementary material of this article. All Python codes used in this study are publicly available at https://github.com/zhanglabtools/ImputeHiFI.
