# Supplementary material for: ImputeHiFI: An Imputation Method for Multiplexed DNA FISH Data by Utilizing Single‐Cell Hi‐C and RNA FISH Data
Source: Adv Sci (Weinh). 2024 Sep 12;11(42):2406364. doi: 10.1002/advs.202406364 (PMC11558076; doi:10.1002/advs.202406364)
Supplement: Supplementary file 1 — Supporting Information [file ADVS-11-2406364-s001.pdf]

ADVANCED SCIENCE
Open Access

Supporting Information

for *Adv. Sci.*, DOI 10.1002/advs.202406364

ImputeHiFI: An Imputation Method for Multiplexed DNA FISH Data by Utilizing Single-Cell Hi-C and RNA FISH Data

Shichen Fan, Dachang Dang, Lin Gao and Shihua Zhang**

Supplementary Information

ImputeHiFI: An Imputation Method for Multiplexed DNA FISH Data by Utilizing Single-Cell Hi-C and RNA FISH Data

Shichen Fan, Dachang Dang, Lin Gao, Shihua Zhang**

S. Fan, L. Gao

School of Computer Science and Technology, Xidian University, Xi'an 710071, China,

E-mail: lgao@mail.xidian.edu.cn

D. Dang

School of Automation, Northwestern Polytechnical University, Xi'an 710072, China

S. Zhang

NCMIS, CEMS, RCSDS, Academy of Mathematics and Systems Science, Chinese Academy of Sciences, Beijing 100190, China;

School of Mathematical Sciences, University of Chinese Academy of Sciences, Beijing 100049, China;

Key Laboratory of Systems Biology, Hangzhou Institute for Advanced Study, University of Chinese Academy of Sciences, Chinese Academy of Sciences, Hangzhou 310024, China.

E-mail: zsh@amss.ac.cn

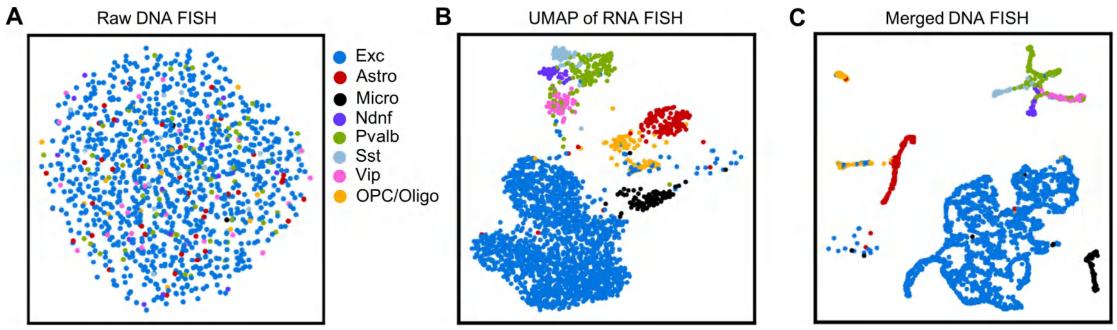

Figure S1. UMAP visualization analysis of Takei et al. DNA FISH and RNA FISH data. (A) UMAP visualization of Takei et al. DNA FISH data. (B) UMAP visualization of Takei et al. RNA FISH data. (C) UMAP visualization of merged Takei et al. DNA FISH data based on K-nearest neighbors of Takei et al. RNA FISH data with K=30.

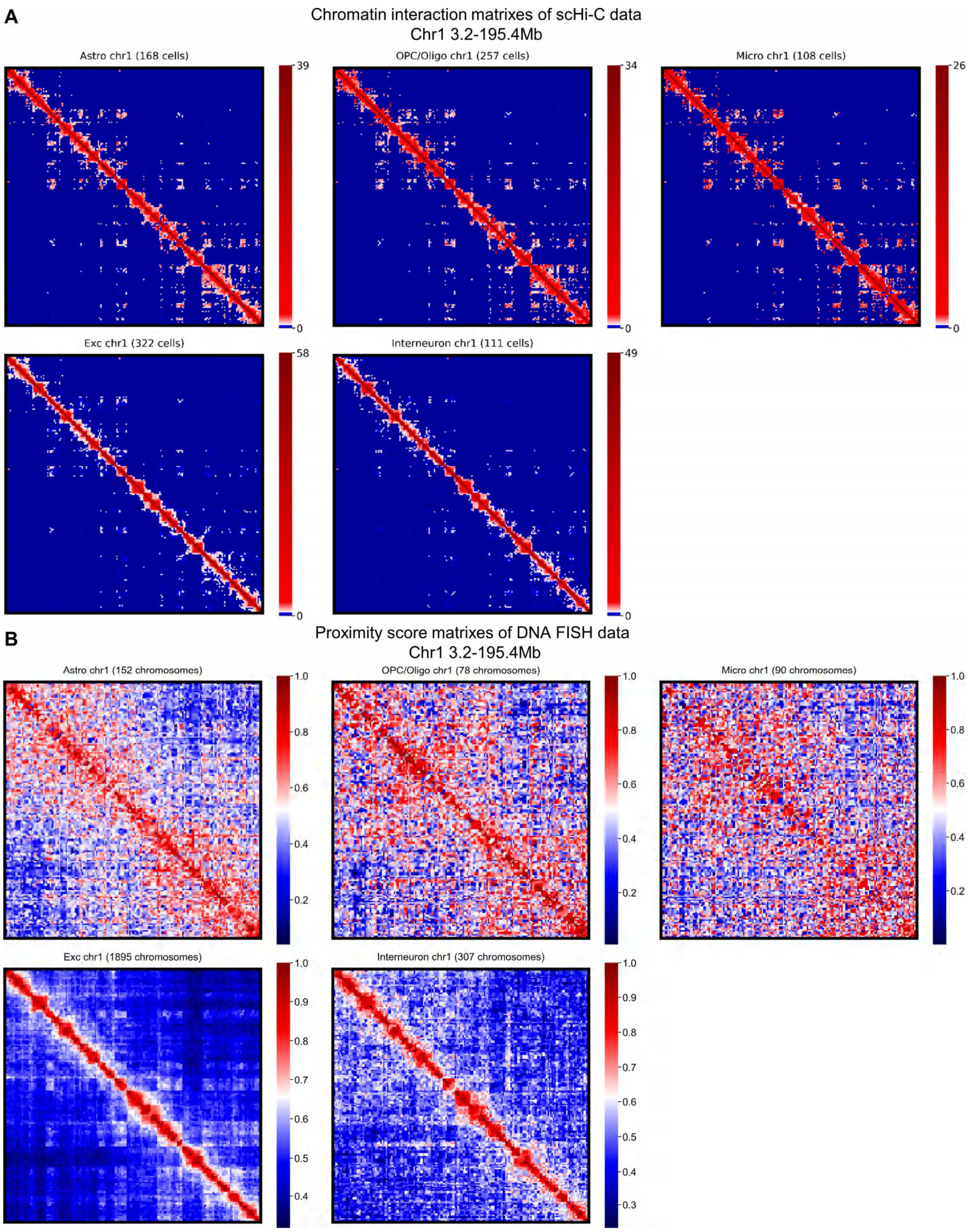

Figure S2. Heatmap of chromatin interaction map from chr1. (A) The heatmap of chromatin interaction matrixes from the Tan et al. scHi-C-seq dataset. In each heatmap, chromosomes from the same cell type are merged by taking the median, and the number of cells is indicated in the title of each subplot. 'Exc' includes 'Cortical L2–5 Pyramidal' and 'Cortical L6 Pyramidal' cells. (B) The heatmap of proximity score matrixes from the Takei et al. dataset. In each heatmap, chromosomes from the same cell type are merged by taking the median, and the number of chromosomes is indicated in the title of each subplot. 'Interneuron' includes 'Pvalb', 'Sst', 'Vip', and 'Ndnf' cells.

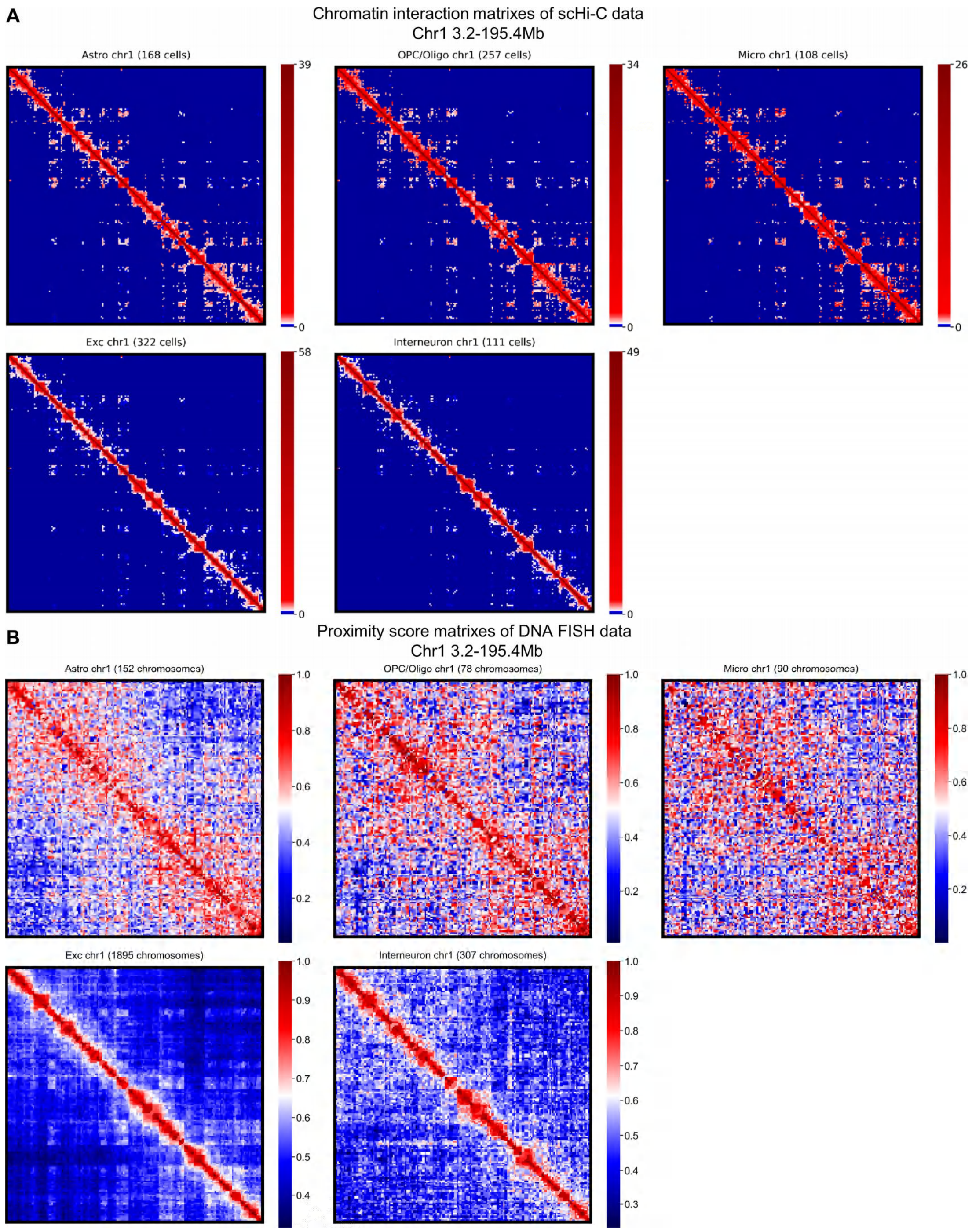

Figure S3. Heatmap of chromatin interaction map from chr7. (A) The heatmap of chromatin interaction matrixes from Tan et al. scHi-C-seq dataset. In each heatmap, chromosomes from the same cell type are merged by taking the median, and the number of cells is indicated in the title of each subplot. ‘Exc’ includes ‘Cortical L2–5 Pyramidal’ and ‘Cortical L6 Pyramidal Cell’. (B) The heatmap of proximity score matrixes from the Takei et al. dataset. In each heatmap, chromosomes from the same cell type are merged by taking the median, and the number of chromosomes is indicated in the title of each subplot. ‘Interneuron’ includes ‘Pvalb’, ‘Sst’, ‘Vip’, and ‘Ndnf’ cells.

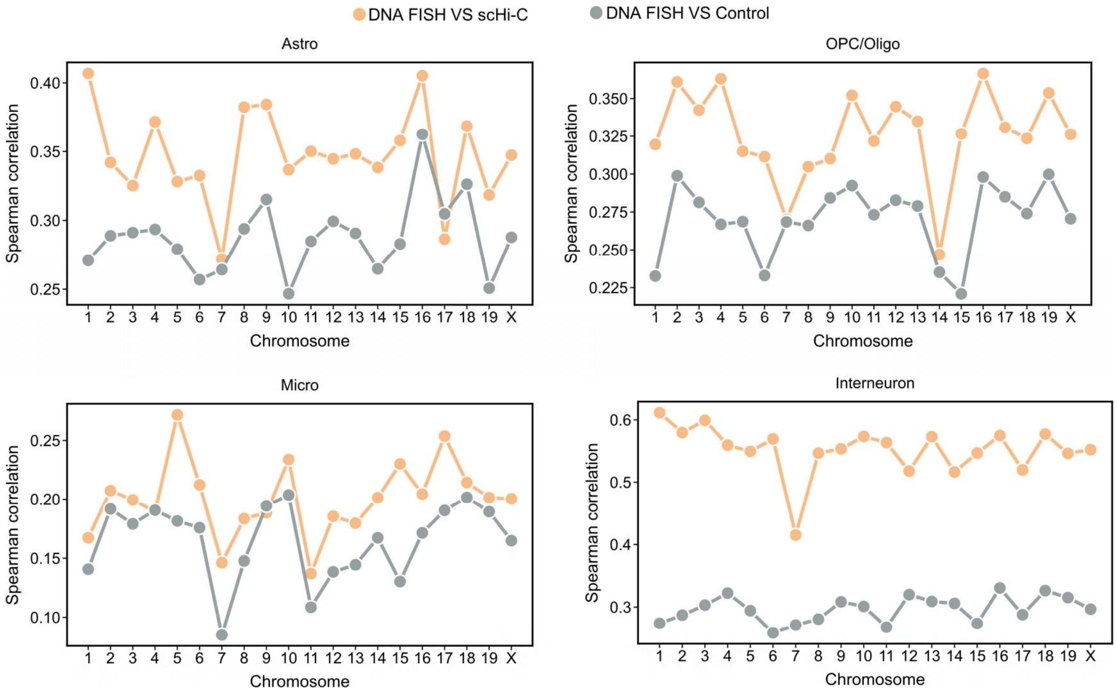

Figure S4. Spearman correlation between the merged 1Mb proximity score maps from multiplexed DNA FISH data and the merged 1Mb chromatin interaction maps from scHi-C for different cell types. Control data are multiplexed DNA FISH data of other cell types.

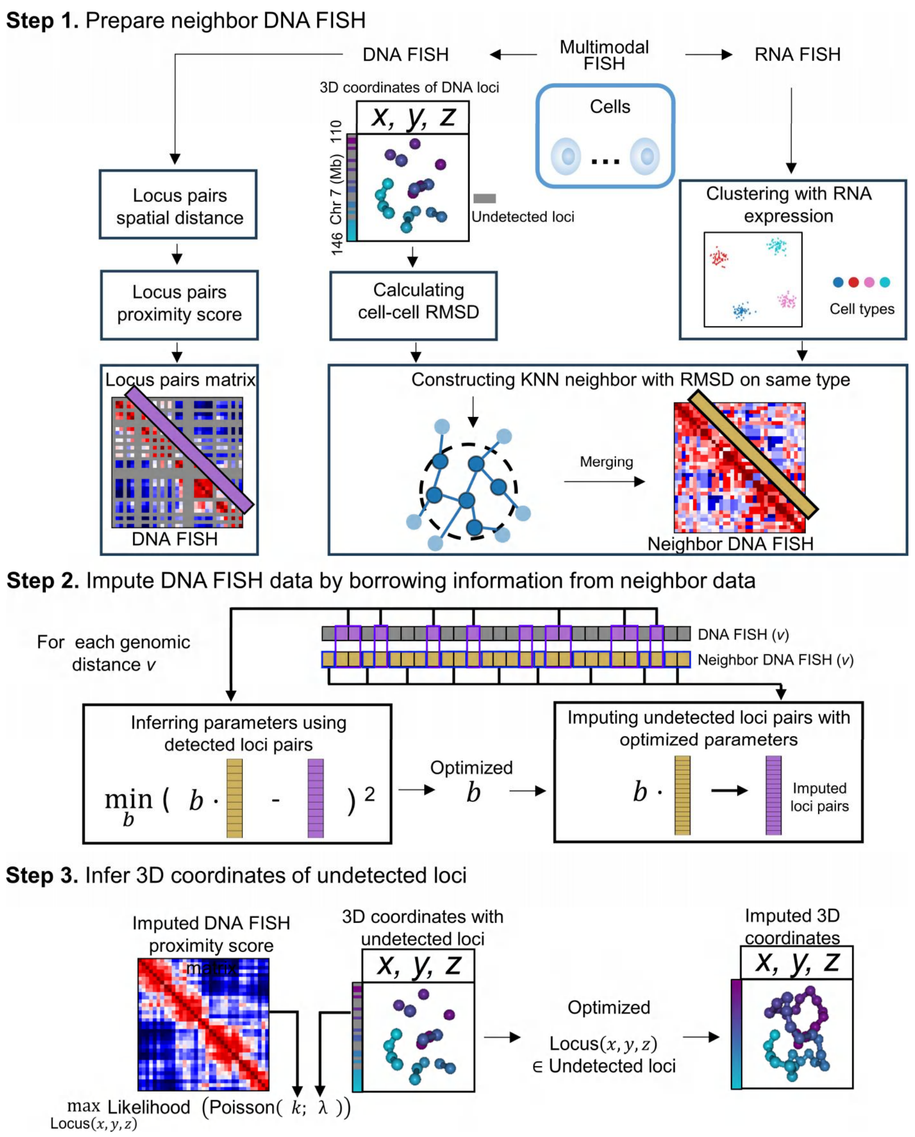

Figure S5. Overview of ImputeHiFI mode 2. To simplify our description, we refer to multiplexed DNA FISH simply as DNA FISH. **Step 1:** Prepare neighbor DNA FISH data. ImputeHiFI uses multimodal DNA FISH and RNA FISH data. ImputeHiFI clusters the RNA FISH data, calculates the RMSD for DNA FISH on the same type of cells to build a cell-type-specific neighbor graph, and merges the neighbor DNA FISH data. **Step 2:** Impute DNA FISH by borrowing information from neighbor data. For each genomic distance v, ImputeHiFI determines neighbor DNA FISH data weight b through the detected loci pairs in DNA FISH data. Then, ImputeHiFI uses b to impute the undetected loci pairs in DNA FISH data. **Step 3:** Infer 3D coordinates of undetected loci. Utilizing the imputed DNA FISH proximity score matrix obtained from step two, along with 3D coordinates containing undetected loci, ImputeHiFI models the proximity score as independent Poisson random variables k, where the 3D coordinates of loci serving as the Poisson parameter λ. By maximizing the likelihood, ImputeHiFI imputes the 3D coordinates of undetected loci.

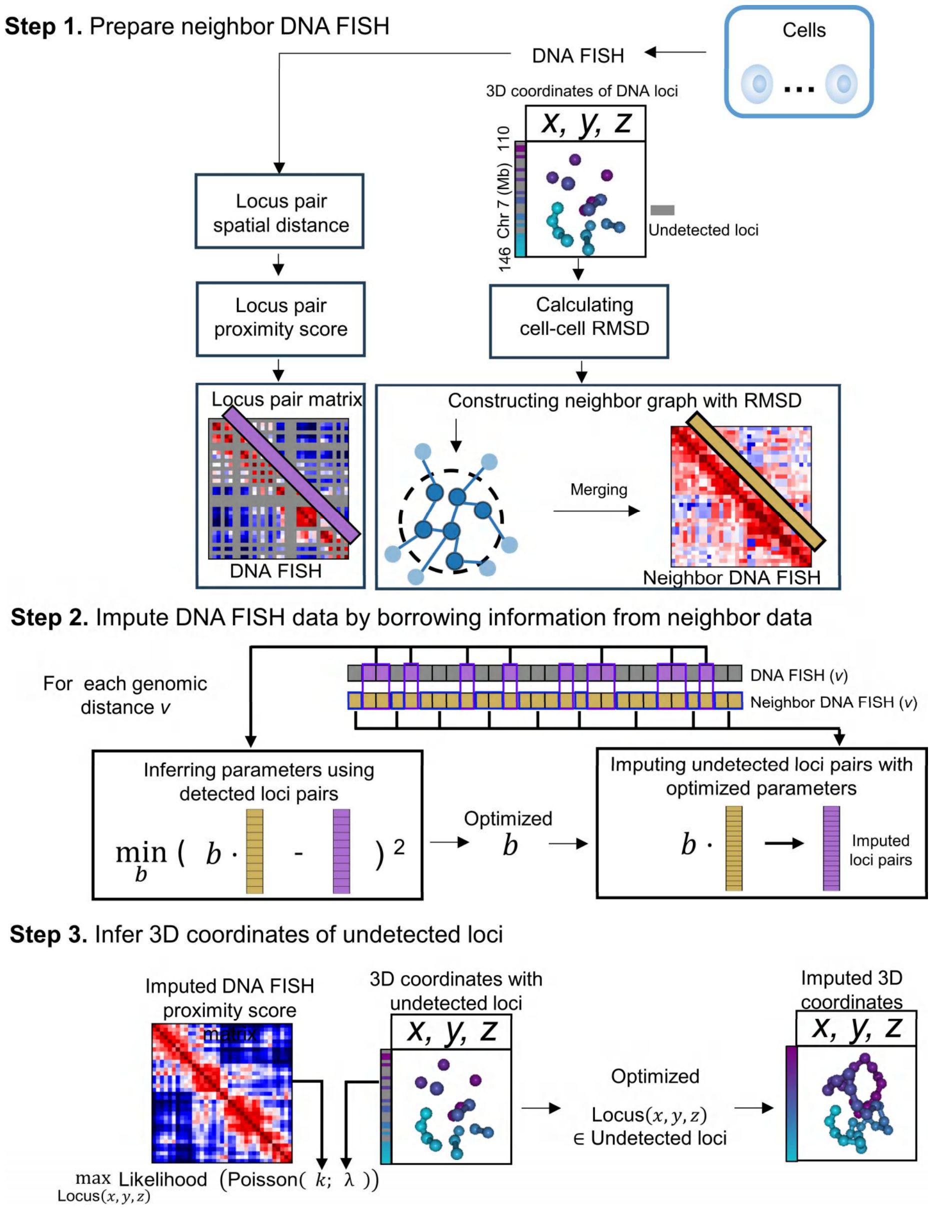

Figure S6. Overview of ImputeHiFI mode 3. Step 1: Prepare neighbor DNA FISH data. ImputeHiFI calculates the RMSD for DNA FISH to build a cell neighbor graph and merges the neighbor DNA FISH data. **Step 2:** Impute DNA FISH by borrowing information from neighbor data. For each genomic distance *v*, ImputeHiFI determines neighbor DNA FISH data weight *b* through the detected loci pairs in DNA FISH data. Then, ImputeHiFI uses b to impute the undetected loci pairs in DNA FISH data. **Step 3:** Infer 3D coordinates of undetected loci. Utilizing the imputed DNA FISH proximity score matrix obtained from step two, along with 3D coordinates containing undetected loci, ImputeHiFI models the proximity score as independent Poisson random variables *k*, where the 3D coordinates of loci serving as the Poisson parameter λ. By maximizing the likelihood, ImputeHiFI imputes the 3D coordinates of undetected loci.

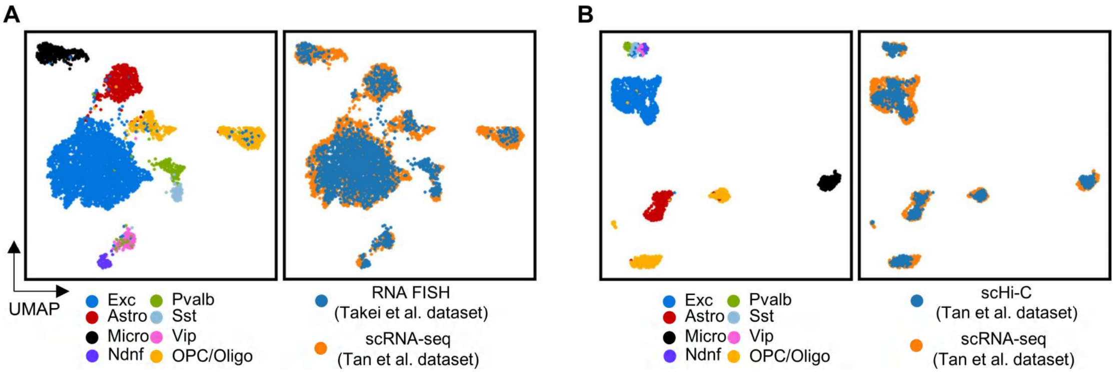

Figure S7. The integration result of MaxFuse and scGAD. (A) The integration of Takei et al. RNA FISH and Tan et al. scRNA-seq data with MaxFuse. Integration score 0.79. (B) The integration of Tan et al. scHi-C and Tan et al. scRNA-seq data with scGAD. Integration score 0.84. Following the recent integration study[1], we defined integration score = 0.6 × biology conservation + 0.4 × omics mixing. Integration score ranges from 0 to 1, and higher values indicate better integration.

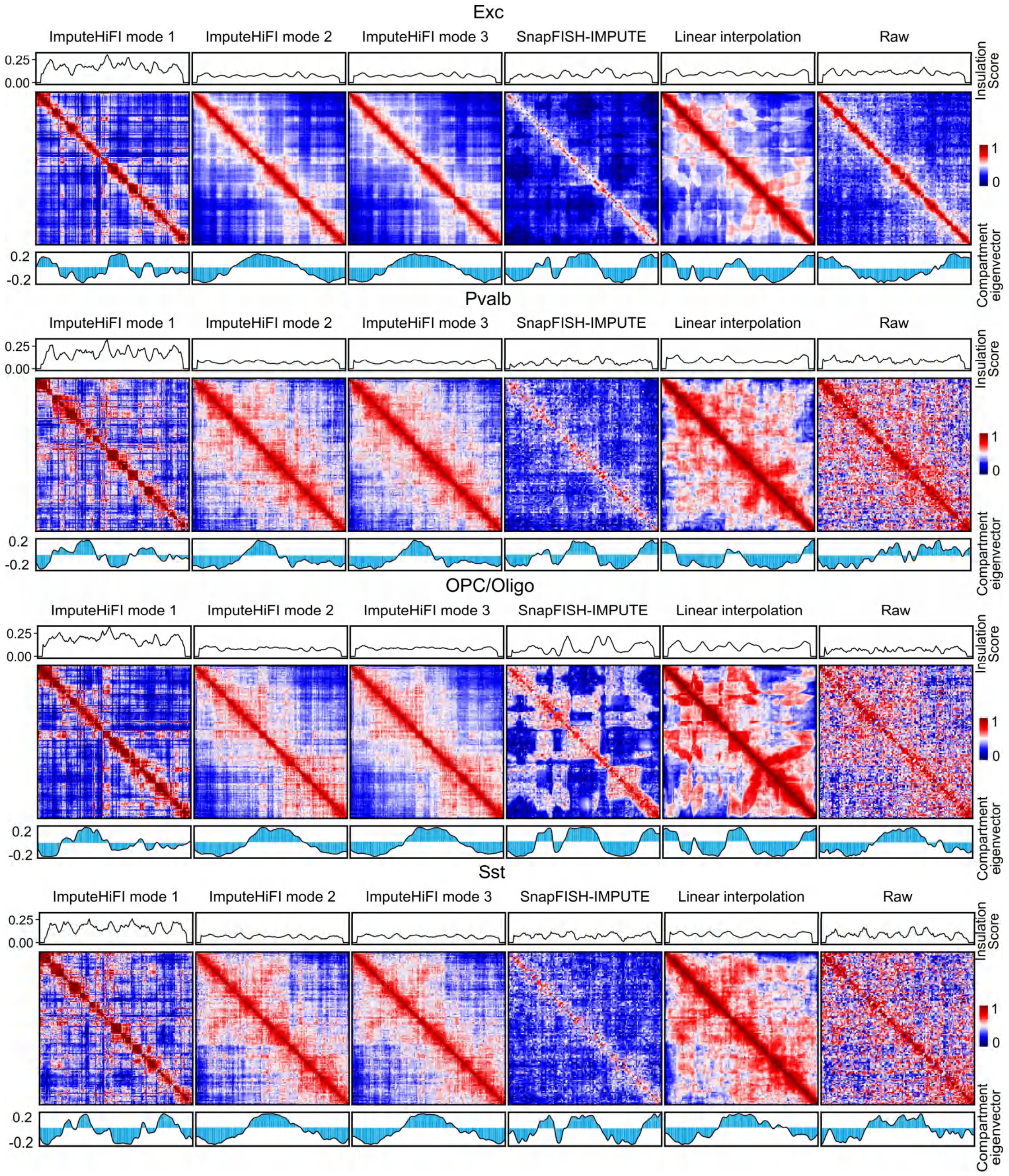

Figure S8. Insulation scores, proximity score heatmaps and compartment eigenvectors of DNA FISH data imputed with different methods and raw data. The probe missing rate is 0.8. DNA FISH data were merged according to cell types. Chr1: 3.2 -195.4 Mb.

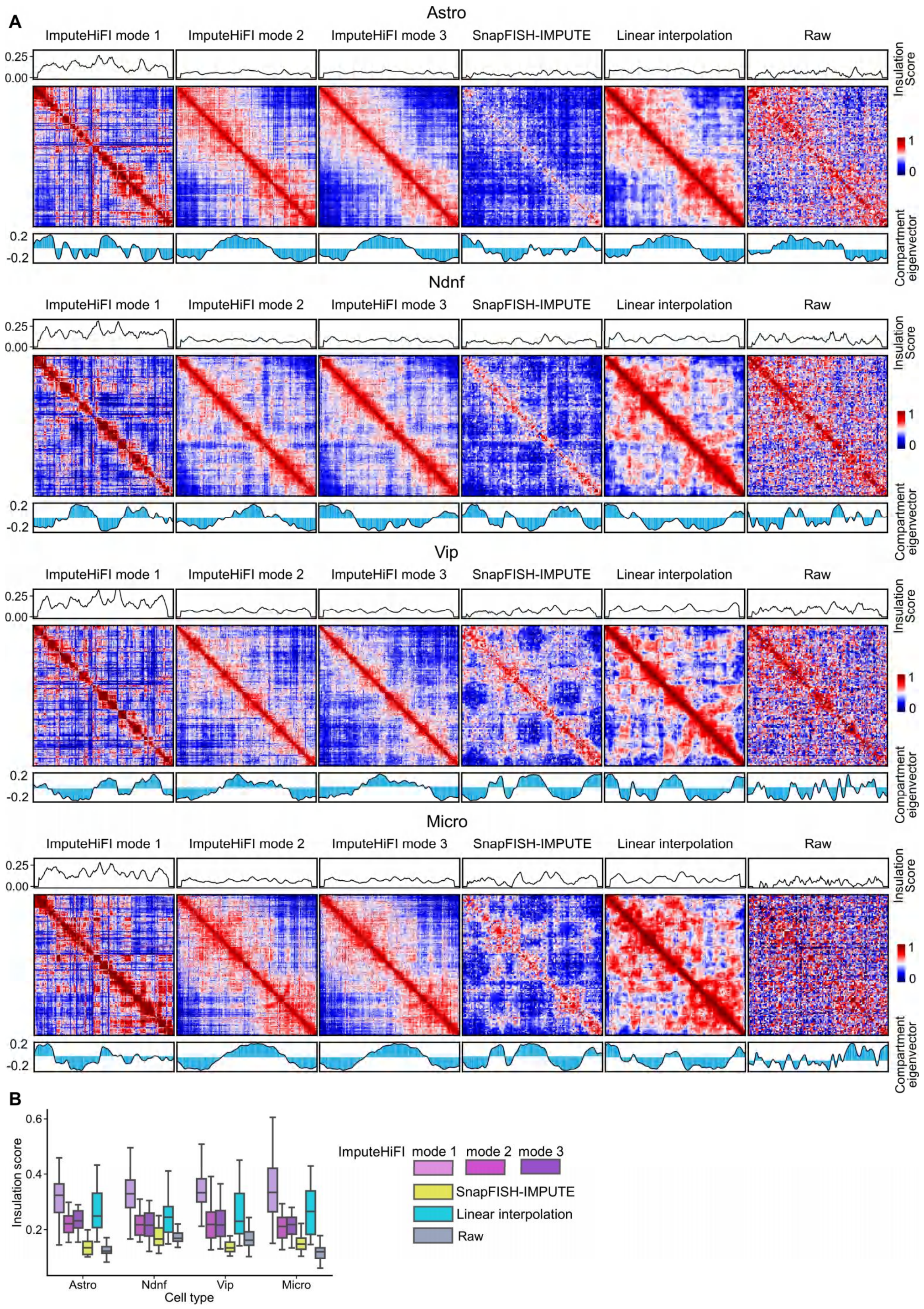

Figure S9. Heatmap of imputed data and boxplot of boundary insulation score. (A) Insulation scores, proximity score heatmaps, and compartment eigenvectors of DNA FISH data imputed with different methods and raw data. The probe missing rate is 0.8. DNA FISH data were merged according to cell types. Chr1: 3.2-195.4 Mb. (B) Boundary insulation scores of DNA FISH data imputed with different methods and raw data. The probe missing rate is 0.8. DNA FISH data were merged according to cell types. The boundaries are the insulation score peaks at the transition point between A and B compartments.

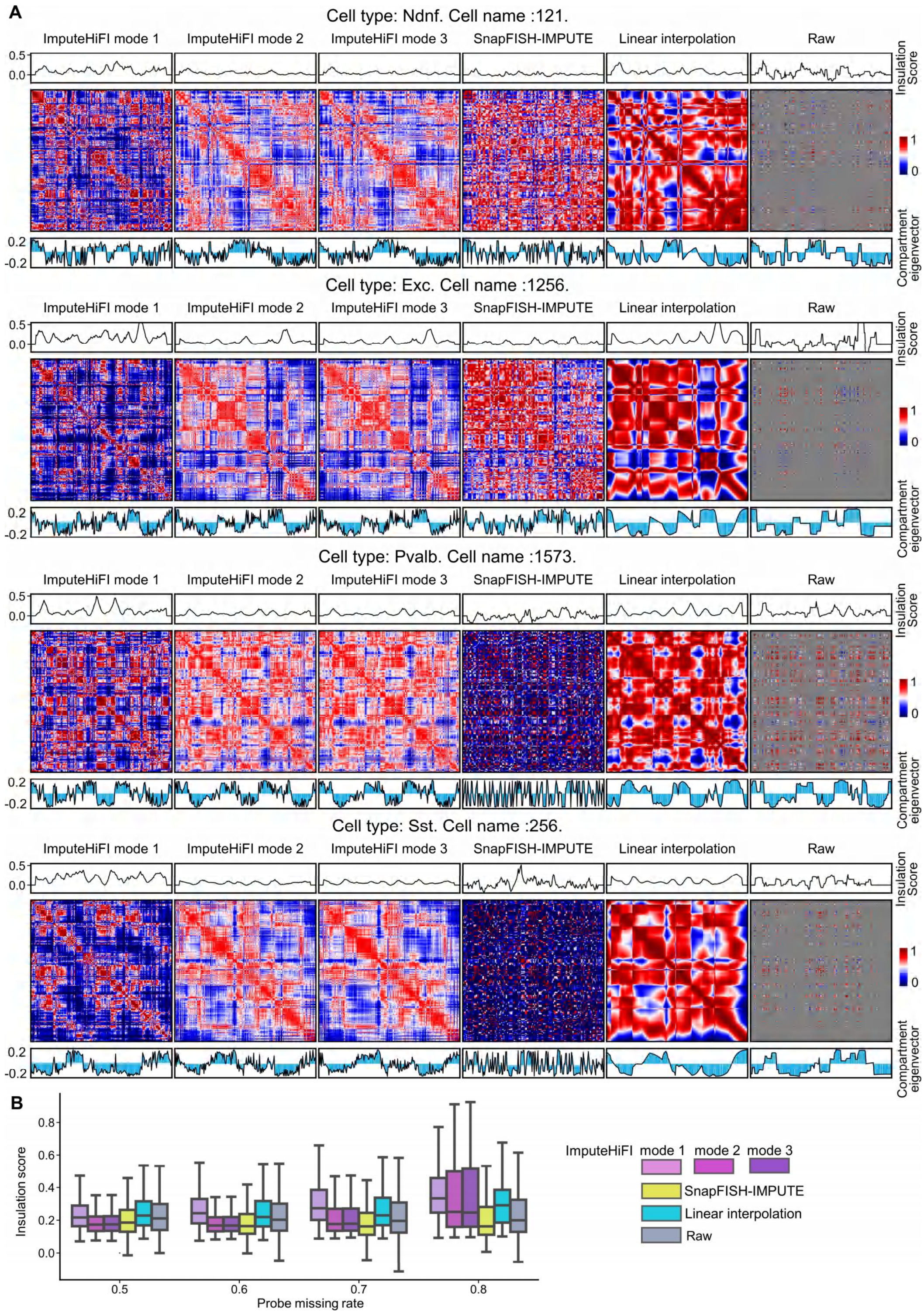

Figure S10. Heatmap of single-cell imputed data and boxplot of boundary insulation score. (A) Single-cell insulation scores, proximity score heatmaps, and compartment eigenvectors of DNA FISH data imputed with different methods and raw data. Chr1: 3.2-195.4 Mb. (B) Single-cell boundary insulation scores of DNA FISH data imputed with different methods and raw data. The boundaries are the insulation score peaks.

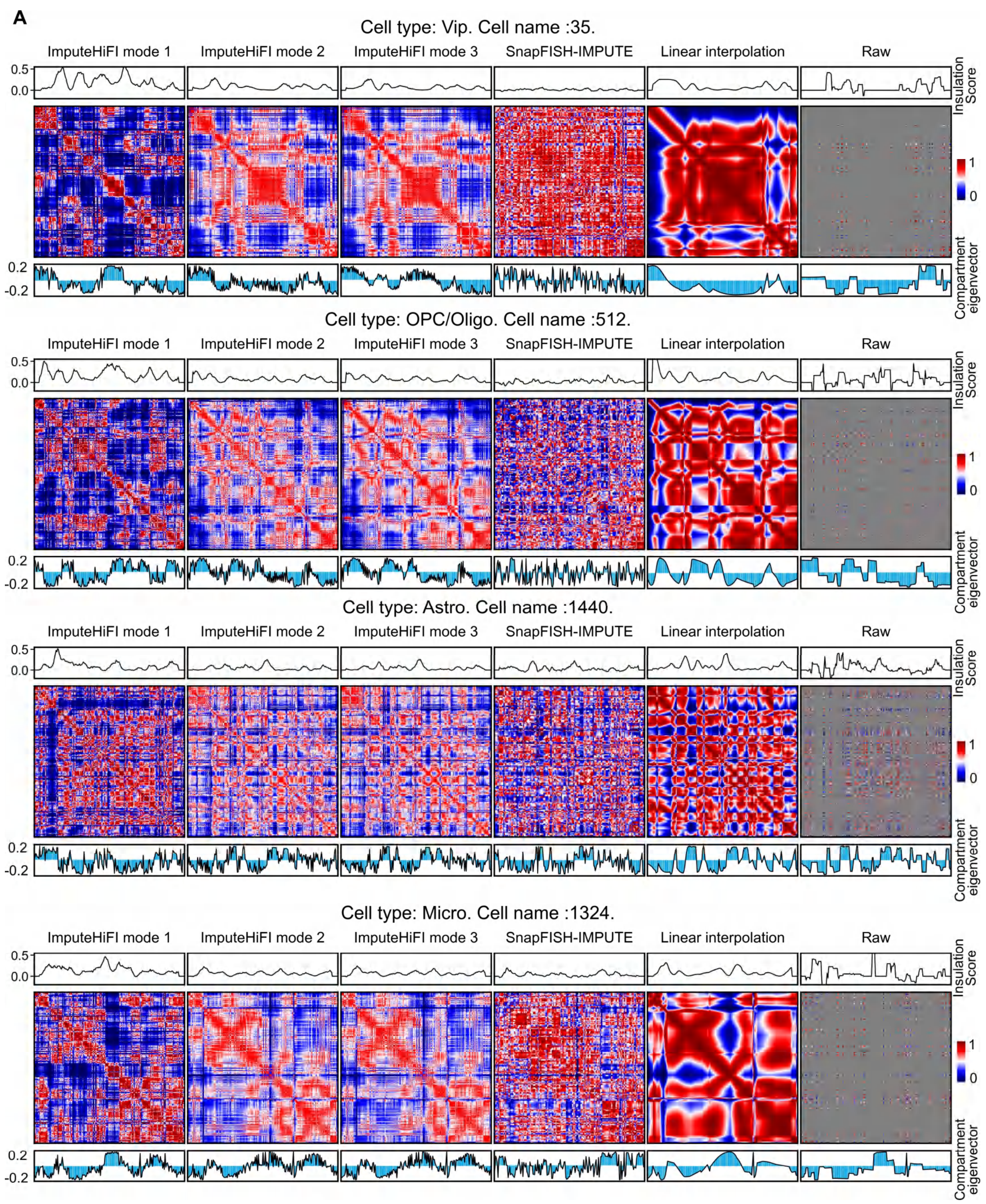

Figure S11. Single-cell insulation scores, proximity score heatmaps, and compartment eigenvectors of DNA FISH data imputed with different methods and raw data. Chr1: 3.2-195.4 Mb.

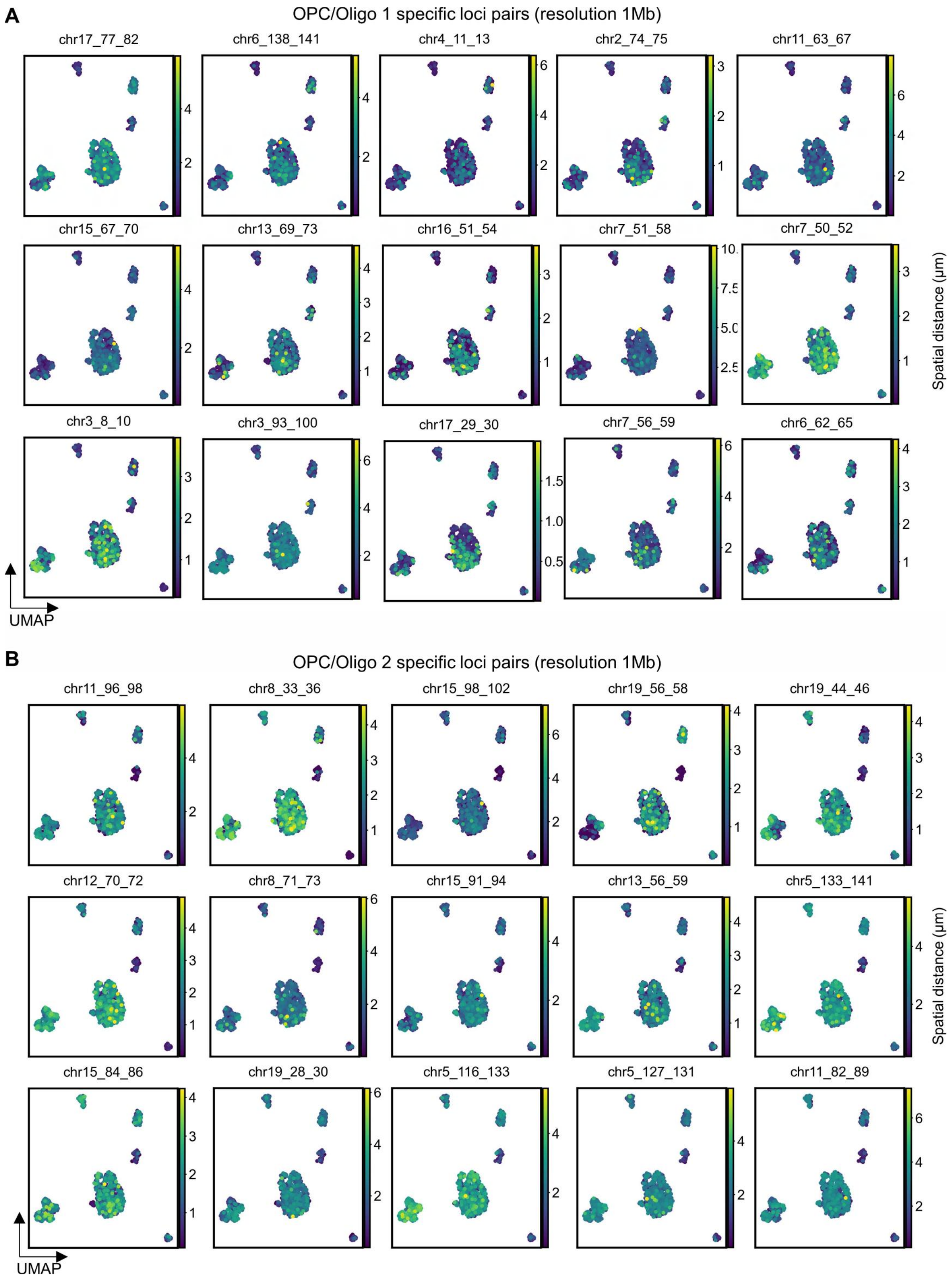

Figure S12. UMAP visualization of the loci pairs showing significant differences between OPC/Oligo 1 and OPC/Oligo 2. (A) The spatial distances of 'OPC/Oligo 1' specific loci pairs from imputed Takei et al. (B) The spatial distances of 'OPC/Oligo 2' specific loci pairs from imputed Takei et al. Multiplexed DNA FISH data (ImputeHiFI mode 1 result).

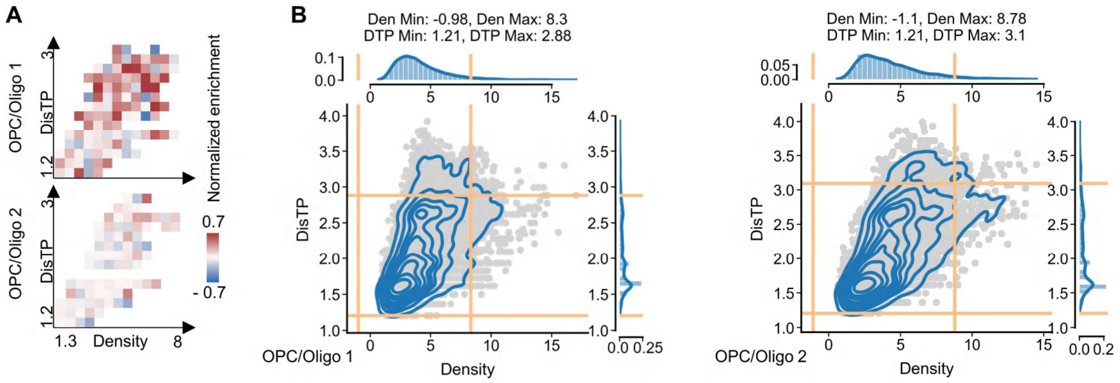

Figure S13. D^2 plots of OPC/Oligo 1 and OPC/Oligo 2. (A) The enrichment scores of oligodendrocyte progenitor marker genes on the D^2 plots of OPC/Oligo 1 and OPC/Oligo 2 (ImputeHiFI mode 1 result). D^2 plot is DNA density and distance to the nuclear periphery (DisTP) 2D matrix plot. (B) 2D histogram and KDE plots of density and DisTP.

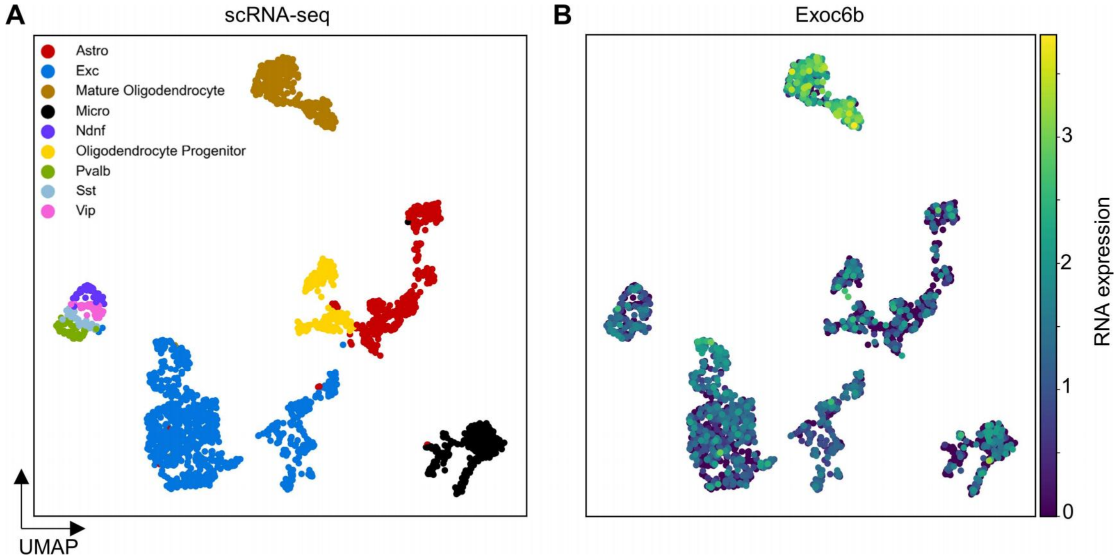

Figure S14. UMAP visualization of the Exoc6b expression in scRNA-seq data. (A) UMAP visualization of the Tan et al. scRNA-seq data. (B) The marker genes expression of the Tan et al. scRNA-seq data.

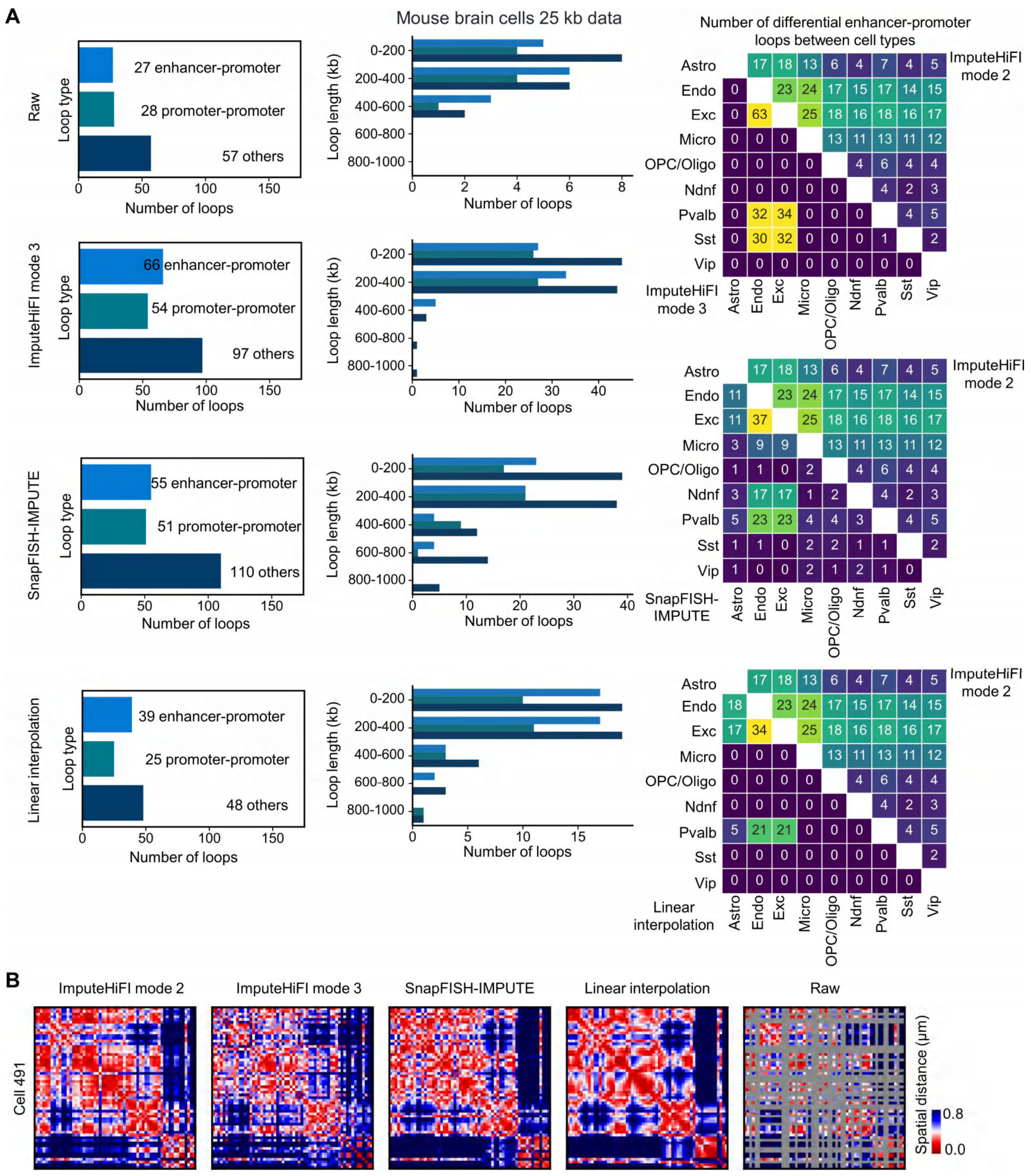

Figure S15. Loop analysis for the Takei et al. multiplexed DNA FISH data on the mouse brain. (A) Left: Loop number for three types of loops. Middle: Loop length is the genomic distance between loop endpoints. The Y-axis represents the loop length interval. Right: Number of differential enhancer-promoter loops between cell types. (B) Single-cell heatmaps of DNA spatial distance matrices. The cell is named ‘491’. Chr6: 49.3-50.9Mb.

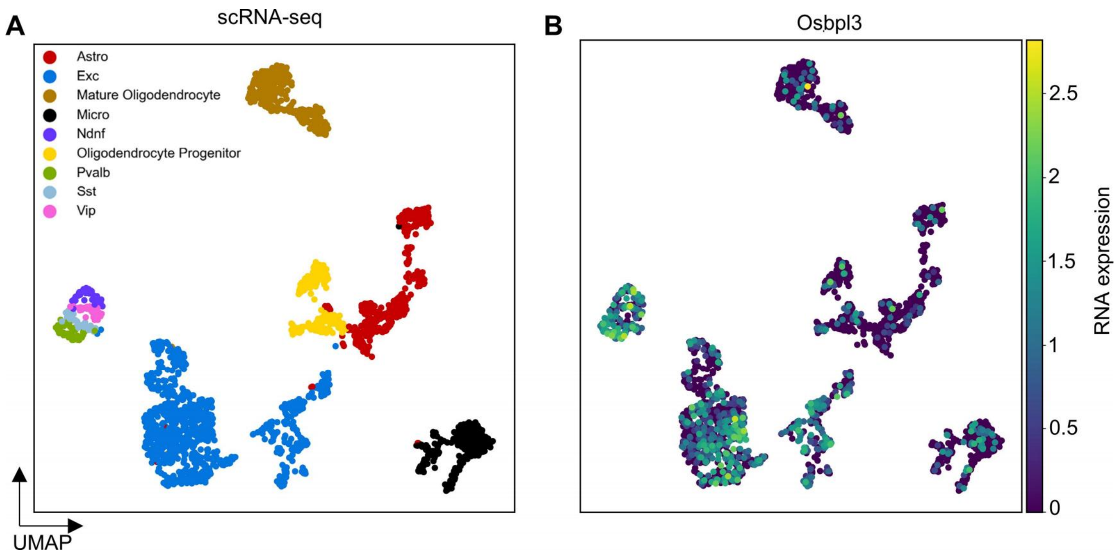

Figure S16. UMAP visualization of the Osbpl3 expression in scRNA-seq data. (A) UMAP visualization of the Tan et al. scRNA-seq data. (B) The marker gene expression of the Tan et al. scRNA-seq data.

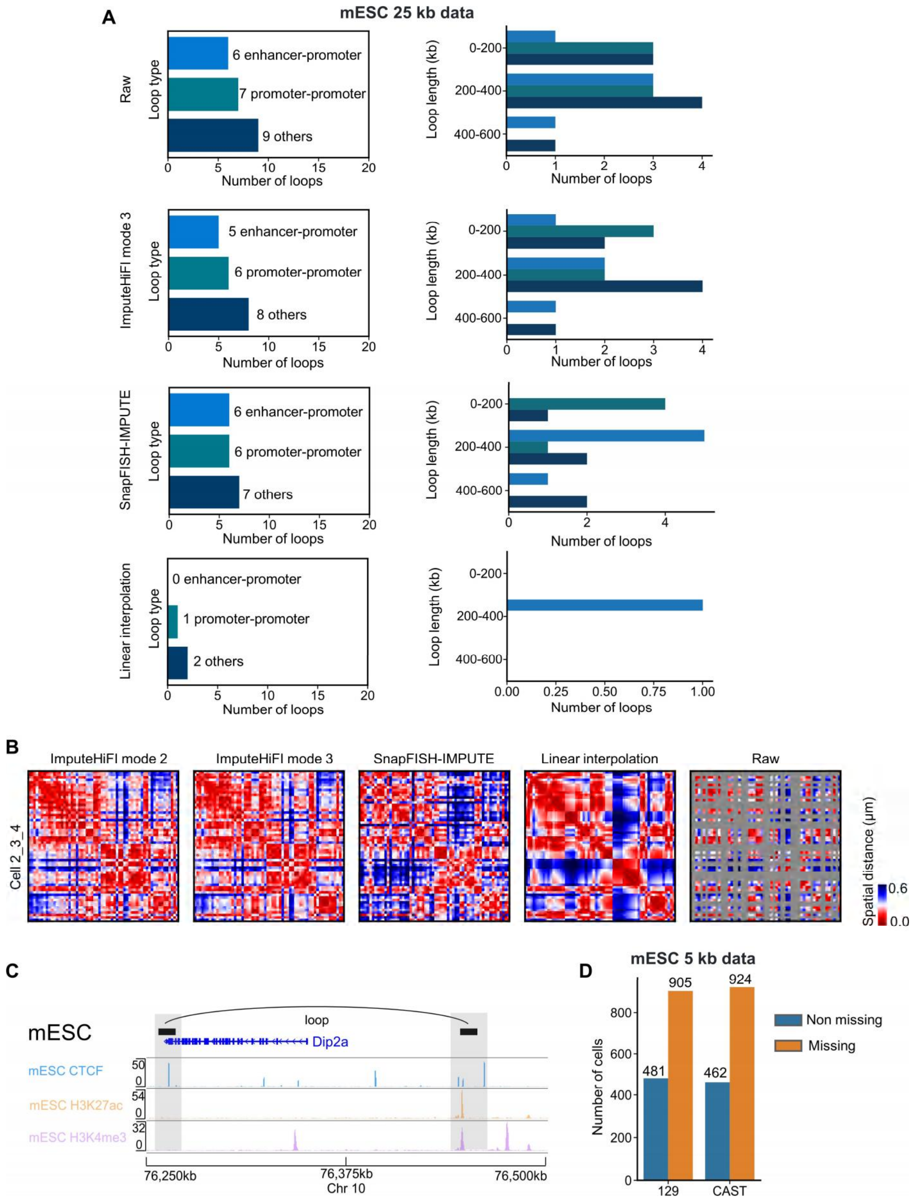

Figure S17. (A-C) Loop result of the Takei et al. multiplexed DNA FISH 25 kb data on the mouse embryonic stem cells. (D) Loop result of Huang et al.'s multiplexed DNA FISH 5 kb data in mouse embryonic stem cells. (A) Left: Loop number for three types of loops. Right: Loop length is the genomic distance between loop endpoints. The Y-axis represents the loop length interval. (B) Single-cell heatmaps of DNA spatial distance matrices. The cell is named '2_3_4'. Chr5: 131.4-132.9Mb. (C) Illustrating the enhancer-promoter loop involving the Dip2a gene, located at chr10: 76,263,051-76,345,260. This loop is consistently identified in the raw and ImputeHiFI mode 1 data. The bottom three tracks are mESC CTCF, mESC H3K27ac, and mESC H3K4me3 ChIP-seq data. (D) The number of cells categorized based on the presence (Non-missing) or absence (Missing) of interactions between the Sox2 gene promoter and its associated super-enhancers.

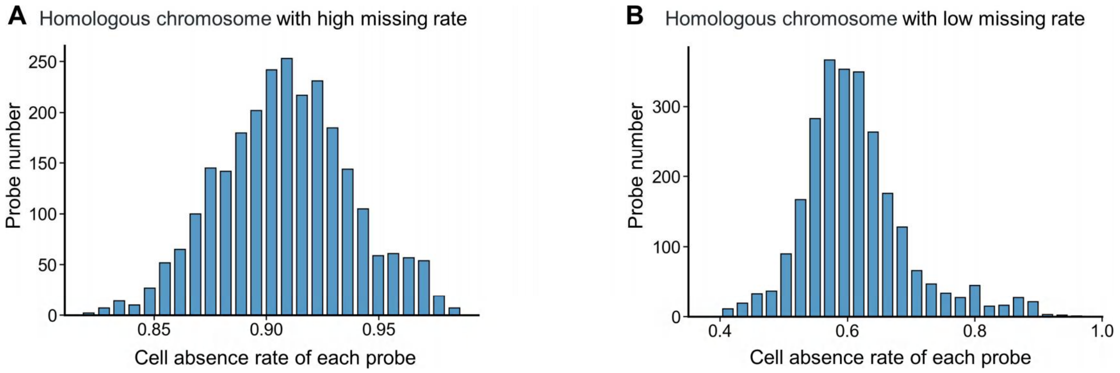

Figure S18. Distributions of probe numbers with varying cell absence rates of each probe. For each probe, the cell absence rate is the number of cells in which it was undetected, divided by the total number of cells. One chromosome of the Takei et al. dataset has two alleles per cell. The homologous chromosomes are categorized into two types with high (A, mean cell absence rate of each probe is 0.9) and low (B, mean cell absence rate of each probe is 0.6) missing rates, respectively.

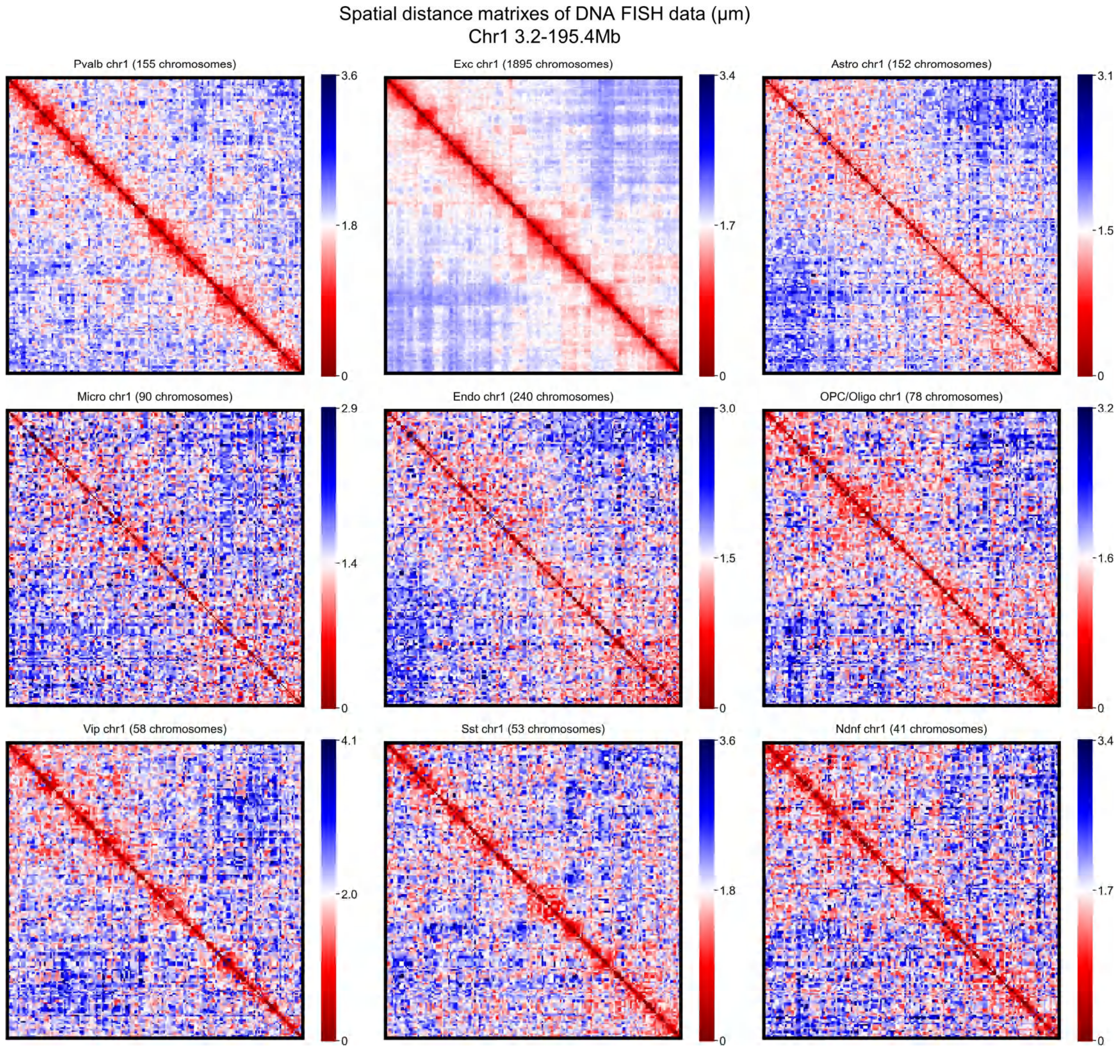

Figure S19. The heatmap of spatial distance matrixes from the Takei et al. dataset. In each heatmap, chromosomes from the same cell type are merged by taking the median, and the number of chromosomes is indicated in the title of each subplot.

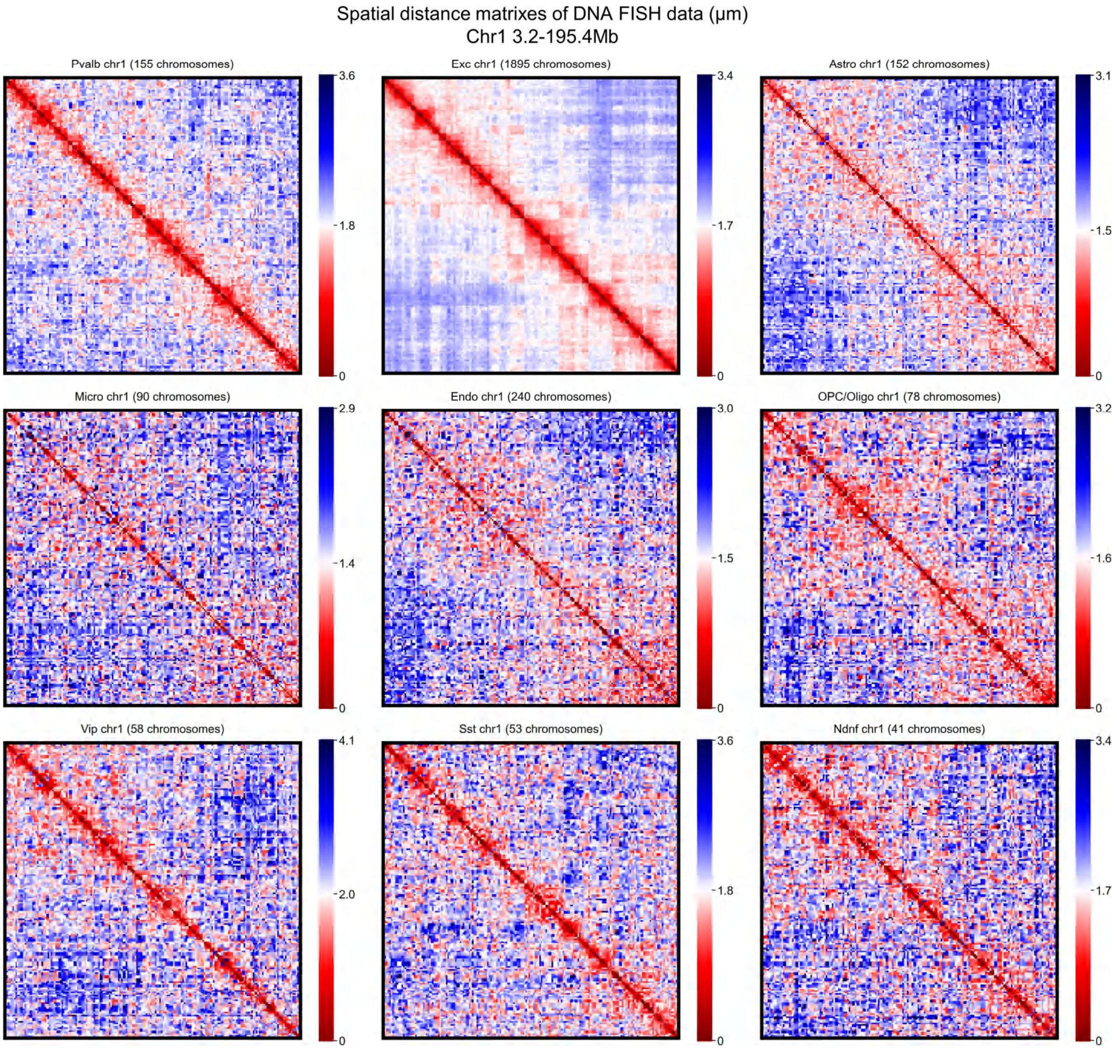

Figure S20. The heatmap of proximity score matrixes from the Takei et al. dataset. In each heatmap, chromosomes from the same cell type are merged by taking the median, and the number of chromosomes is indicated in the title of each subplot.

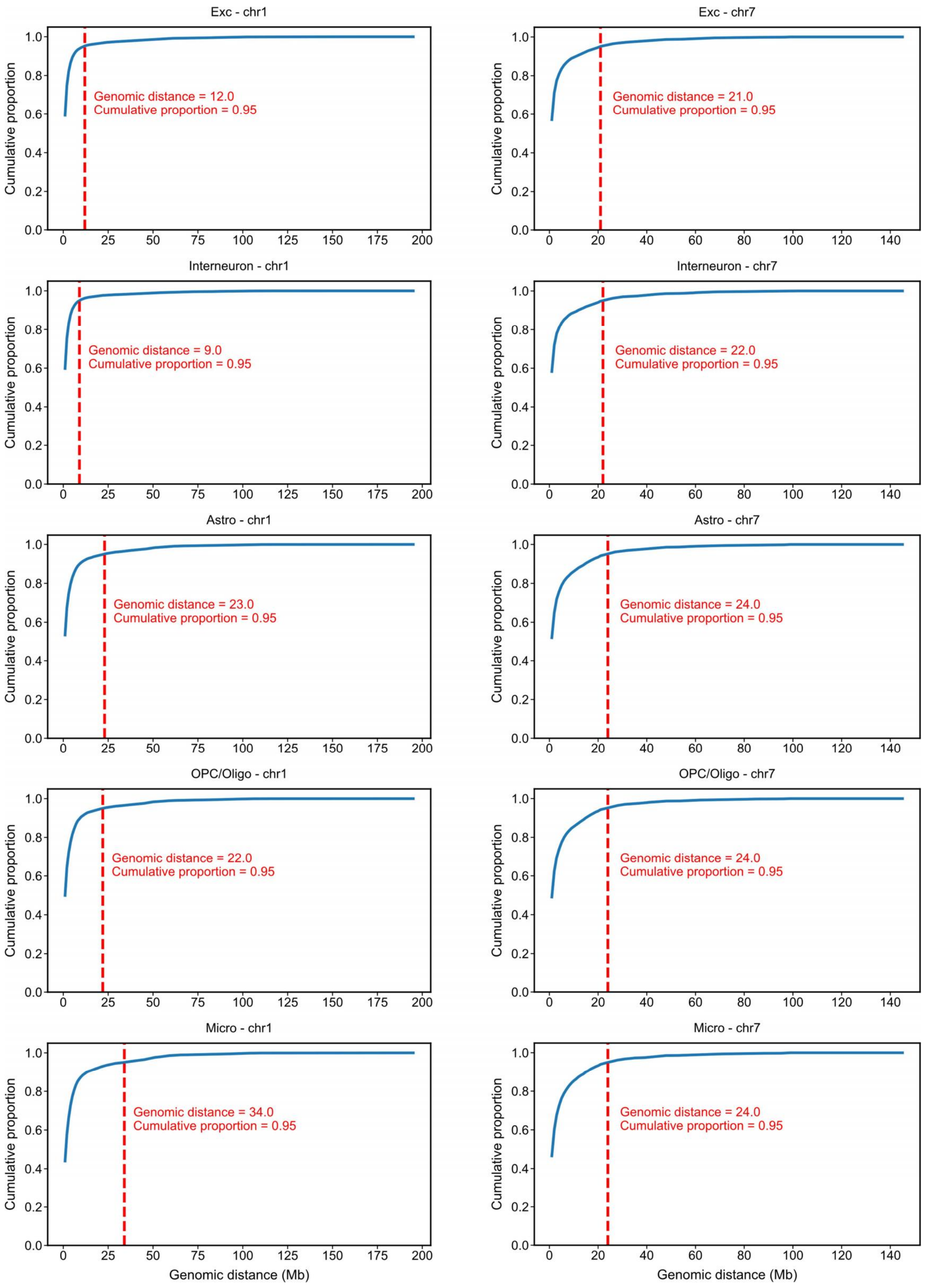

Figure S21. The cumulative proportion of the values along the diagonal relative to the total sum of all values in the Hi-C matrix as the diagonal progresses (Tan et al. scHi-C-seq dataset). Different diagonals in the Hi-C matrix represent different genomic distances.

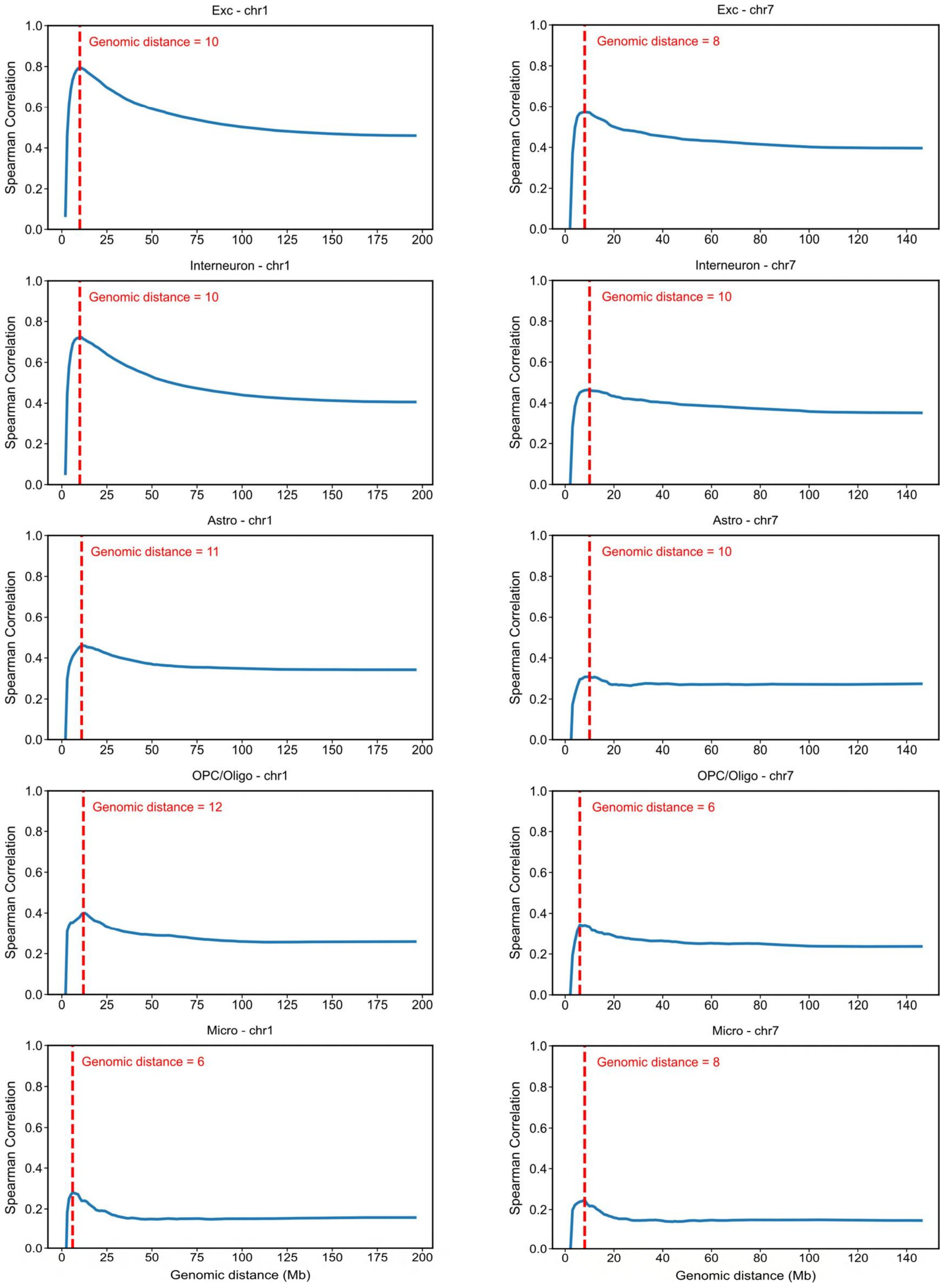

Figure S22. Spearman correlation coefficient between merged single-cell Hi-C data (Tan et al. scHi-C-seq dataset) and merged multiplexed DNA FISH data (Takei et al. dataset) within a specific genomic distance range.

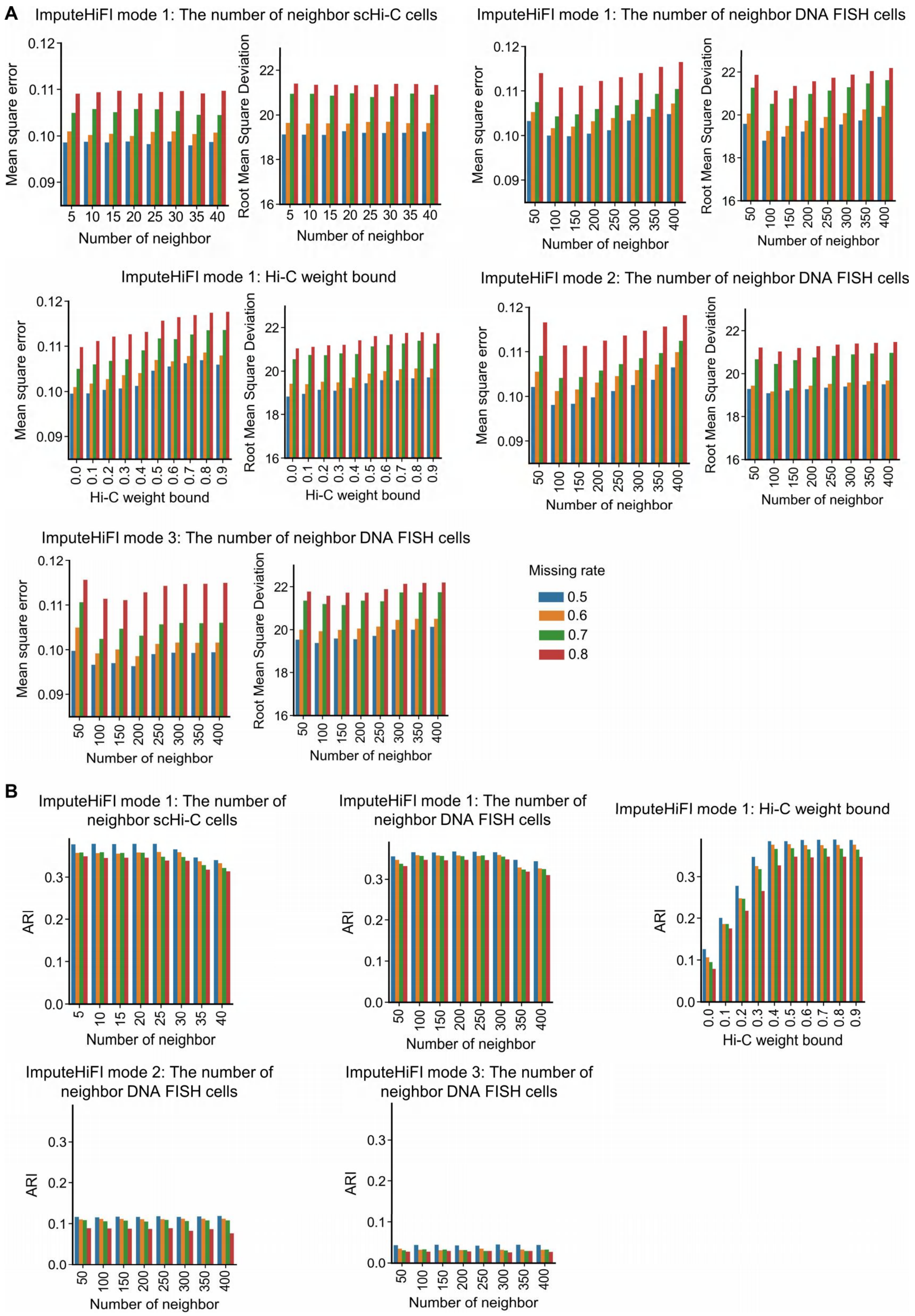

Figure S23. Parameter selection for simulated and real data. (A) The impact of different parameters on results in simulated data. (B) The impacts of different parameters on results in real data (Takei et al.'s DNA seqFISH+ 1Mb data on the mouse brain cells).

References

[1] Z.-J. Cao, G. Gao, *Nat. Biotechnol.* **2022**, *40* (10), 1458.
